# Raf kinase inhibitor protein mediates myocardial fibrosis under conditions of enhanced myocardial oxidative stress

**DOI:** 10.1007/s00395-018-0700-3

**Published:** 2018-09-06

**Authors:** Andrey Kazakov, Rabea A. Hall, Christian Werner, Timo Meier, André Trouvain, Svetlana Rodionycheva, Alexander Nickel, Frank Lammert, Christoph Maack, Michael Böhm, Ulrich Laufs

**Affiliations:** 1 Klinik für Innere Medizin III, Kardiologie, Angiologie und Internistische Intensivmedizin, Universität/Universitätsklinikum des Saarlandes, Kirrberger Strasse 100, IMED, 66421 Homburg, Germany; 2 Klinik für Innere Medizin II, Gastroenterologie, Hepatologie, Endokrinologie, Diabetologie und Ernährungsmedizin, Universität/Universitätsklinikum des Saarlandes, Kirrberger Strasse 77, 66421 Homburg, Germany; 3 Klinik für Thorax- und Herz-Gefäßchirurgie, Universität/Universitätsklinikum des Saarlandes, Kirrberger Strasse 57, 66421 Homburg, Germany; 4 Deutsches Zentrum für Herzinsuffizienz, Universitätsklinikum Würzburg, am Schwarzenberg 15, A15, 97078 Würzburg, Germany; 5 Klinik und Poliklinik für Kardiologie, Universitätsklinikum Leipzig, Liebigstrasse 20, 04103 Leipzig, Germany

**Keywords:** RKIP, Cardiac fibrosis, Oxidative stress, Nrf2

## Abstract

Fibrosis is a hallmark of maladaptive cardiac remodelling. Here we report that genome-wide quantitative trait locus (QTL) analyses in recombinant inbred mouse lines of C57BL/6 J and DBA2/J strains identified Raf Kinase Inhibitor Protein (RKIP) as genetic marker of fibrosis progression. C57BL/6 N-RKIP^−/−^ mice demonstrated diminished fibrosis induced by transverse aortic constriction (TAC) or CCl_4_ (carbon tetrachloride) treatment compared with wild-type controls. TAC-induced expression of collagen Iα2 mRNA, Ki67^+^ fibroblasts and marker of oxidative stress 8-hydroxyguanosine (8-dOHG)^+^ fibroblasts as well as the number of fibrocytes in the peripheral blood and bone marrow were markedly reduced in C57BL/6 N-RKIP^−/−^ mice. RKIP-deficient cardiac fibroblasts demonstrated decreased migration and fibronectin production. This was accompanied by a two-fold increase of the nuclear accumulation of nuclear factor erythroid 2-related factor 2 (Nrf2), the main transcriptional activator of antioxidative proteins, and reduced expression of its inactivators. To test the importance of oxidative stress for this signaling, C57BL/6 J mice were studied. C57BL/6 J, but not the C57BL/6 N-strain, is protected from TAC-induced oxidative stress due to mutation of the nicotinamide nucleotide transhydrogenase gene (Nnt). After TAC surgery, the hearts of Nnt-deficient C57BL/6 J-RKIP^−/−^ mice revealed diminished oxidative stress, increased left ventricular (LV) fibrosis and collagen Iα2 as well as enhanced basal nuclear expression of Nrf2. In human LV myocardium from both non-failing and failing hearts, RKIP-protein correlated negatively with the nuclear accumulation of Nrf2. In summary, under conditions of Nnt-dependent enhanced myocardial oxidative stress induced by TAC, RKIP plays a maladaptive role for fibrotic myocardial remodeling by suppressing the Nrf2-related beneficial effects.

**Electronic supplementary material:**

The online version of this article (10.1007/s00395-018-0700-3) contains supplementary material, which is available to authorized users.

## Introduction

Fibrotic remodeling of the myocardium is a hallmark of frequent cardiac pathologies such as heart failure and atrial fibrillation [42]. The fibrotic modulation of the extracellular matrix is controlled by the interaction between cardiac fibroblasts and other cardiac and extracardiac cells [12, 17, 42]. Pathologic left ventricular afterload occurs in conditions such as hypertension, aortic valve stenosis or heart failure and causes interstitial and replacement cardiac fibrosis [21]. Cardiac fibrogenesis is modified by the genetic background and the myocardial redox state, however, important aspects of these interactions remain to be elucidated [29]. In the myocardium, reverse-mode nicotinamide nucleotide transhydrogenase (Nnt) represents the dominant source of increased mitochondrial production of reactive oxygen species (ROS) during pressure overload [29]. Due to a mutation of the *Nnt* gene, the inbred mouse strain C57BL/6 J is protected from pressure overload-induced oxidative stress and heart failure. In contrast, C57BL/6 N mice demonstrate enhanced ROS production and marked cardiac remodeling and failure in pressure overload [29]. We took advantage of these mice to study the complex role of ROS during fibrogenesis.

To identify regulators of myocardial fibrogenesis, genome-wide quantitative trait locus (QTL) analyses were performed. QTL analysis is a powerful method of systemic genetics for the detection of genetic loci linked to trait variation revealing the underlying genetic mechanisms of complex traits such as a fibrosis [13]. 26 BxD recombinant inbred mouse lines, the parental strains C57BL/6 J and DBA/2 J and the F1 hybrids were examined [13]. Our studies identify *Raf Kinase Inhibitor Protein (RKIP)* as a genetic marker of individual fibrosis progression. *RKIP*, also known as *Phosphatidylethanolamine Binding Protein I (PEBP*-*I)*, has been shown to regulate myocardial hypertrophy and inotropy [3, 11, 14, 38]. Based on the QTL analysis, we performed a detailed characterization of RKIP-dependent signalling in C57BL/6 N and J mice that revealed a novel maladaptive mechanism for fibrotic cardiac remodeling under conditions of enhanced myocardial oxidative stress.

## Methods

A more detailed description of the materials and methods is provided in the Supplementary material.

### Animals and experimental design

C57BL/6 N (N) albino wild-type (WT) mice (Charles River, Germany), *RKIP*^−*/*−^ C57BL/6 N albino mice (Brown University, Rhode Island, USA), C57BL/6 J (J) wild-type mice (The Jackson Laboratory, USA) and *RKIP*^−*/*−^C57BL/6 J mice (provided by Kristina Lorenz, Leibniz-Institut für Analytische Wissenschaften, Dortmund, Germany) were used [38, 41]. For QTL analysis C57BL/6 J, DBA/2 J, B6D2 F1 hybrids and BxD lines were obtained from The Jackson Laboratory or from Oak Ridge Laboratory, USA, and bred in the facility of the Neurobsik consortium at the VU University Amsterdam [13].

The studies were approved by the Animal Ethics Committee of the Universities of Saarland and Würzburg and conformed to the Guide for the Care and Use of Laboratory Animals published by the Association for Assessment and Accreditation of Laboratory Animal Care (NRC 2011). Transverse aortic constriction (TAC) and treatment with carbon tetrachloride (CCl_4_) were performed to induce replacement and interstitial fibrosis as described [13, 17]. For surgery and left ventricle (LV)-pressure measurements animals were intraperitoneally anaesthetized with 100 mg/kg body weight ketaminehydrochloride (Ketanest^®^, Pfizer, Germany) and 10 mg/kg body weight xylazinehydrochloride (Rompun^®^ 2%, Bayer, Germany). Anaesthetic monitoring was performed by testing of rear foot reflexes before and during procedures, observation of respiratory pattern, mucous membrane color, and responsiveness to manipulations throughout the procedures. Experimental and control mice from our work group were sacrificed by i.p. injection of ketamine (1 g/kg body weight) and xylazine (100 mg/kg) and hearts were rapidly excised.

### QTL analysis

All fibrosis trait data were uploaded into the GeneNetwork database. Pearson’s correlation was used to correlate fibrosis data among themselves and to BxD phenotypes. The identification and mapping of phenotypic QTLs was performed by linking trait data to genotypes at known genetic marker loci. For the identification of single QTLs, interval mapping analyses were performed across all chromosomes [13].

### Human heart samples

The samples of failing human hearts were obtained from the left ventricle (LV) of patients undergoing heart transplantation due to end-stage heart failure (NYHA IV) (*n* = 14) and of patients with aortic stenosis undergoing aortic valve replacement (NYHA II-III) (*n* = 7). Samples from eight non-failing donor hearts that could not be transplanted for technical reasons served as controls. All samples were obtained with written and informed consent from patients or families of organ donors. The analysis was approved by the Ethics Committee of the Universität des Saarlandes and conformed to the Declaration of Helsinki.

### Circulating fibroblasts

Circulating fibroblasts (fibrocytes) in the peripheral blood and bone marrow were detected by flow cytometry [17]. Cells from blood and bone marrow (BM) were double labelled with the following antibodies: biotin-conjugated anti-collagen I (Rockland, Germany) with streptavidin–fluorescein isothiocyanate (FITC) (Vector Laboratories, USA) and CD45-allophycocyanin (APC) (Pharmingen, Germany). The viable lymphocyte population was examined by flow cytometry (BD FACS Calibur™ instrument and BD CellQuest™ software) [17].

### Cell culture and migration assay

Adult mouse cardiac fibroblasts were isolated from the mouse hearts and migration assay was performed by modified Boyden chambers filled with the medium containing stromal derived factor 1 (SDF-1) [17]. The number of migrated fibroblast immunostained for intracellular fibronectin was evaluated by fluorescence microscopy [17].

### Transfection experiments

Transfection of the second passage of adult cardiac fibroblasts from WT C57BL/6 N mice with siRNA *Pbp* (RKIP) 2 (SI01370194, Qiagen, USA) or with scrambled control RNA (con RNA) (1022076, Qiagen, Germany) using Lipofectamine RNAiMAX (13778-030, Thermo Fisher Scientific, Germany) and Opti-MEM reduced serum medium (31985-070, Thermo Fisher Scientific, Germany) was performed according to the manufacturer´s instructions. After 24 h of transfection ACF were treated with 1 µM angiotensin II (Sigma-Aldrich, Germany) for 5 h. Untreated ACF were used as controls. Cells were harvested for preparation of nuclear and cytosolic protein fractions.

### Immunofluorescence analysis

To detect fibroblasts, cardiomyocytes, cycling cells, CXCR4, platelet-derived growth factor receptor alpha (PDGFRα) and level of oxidative stress immunostaining on 3 µm paraffin sections of the LV were performed using heat-mediated antigen retrieval with citraconic anhydride solution followed by overnight incubation at 4 °C with the first antibody and incubation with the appropriate secondary antibody at 37 °C for 1 h [17, 18].

### Apoptosis

Apoptosis was quantitated using 3 µm thin sections of formalin-fixed heart sections and the ApopTag Peroxidase in situ Oligo Ligation Kit (Millipore, Germany). To evaluate apoptosis rate in cardiomyocytes, non-cardiomyocytes, and fibroblasts specific immunostainings were performed after the apoptosis assay [17, 18].

### Gene expression

RNA and proteins were isolated from the left ventricle. Gene expression was assessed by the semi-quantitative reverse transcriptase-polymerase chain reaction (RT-PCR), the real-time quantitative RT-PCR and western blot [17, 18].

### Malondialdehyde concentrations

Lipid peroxidation was performed using the ALDetect Lipid Peroxidation Assay Kit (Enzo Life Science, Germany) to detect the concentrations of malondialdehyde (MDA) according to the manufacturer´s instructions [29].

### Statistical analysis

Results are presented as mean ± standard error of the mean (SEM). Mann–Whitney-test was used for the comparison of two groups. For experiments with more than two groups, one-way ANOVA with Fisher LSD post hoc test was used. Correlations were assessed with Spearman analysis. Values of *p* < 0.05 were considered significant. SPSS version 18.0 (SPSS Inc., Chicago, Illinois) and Microsoft Excel were used for statistical calculations and graphics creation.

## Results

### QTL analyses identify Raf Kinase Inhibitor Protein as a genetic marker of fibrosis progression

**Fig. 1:**
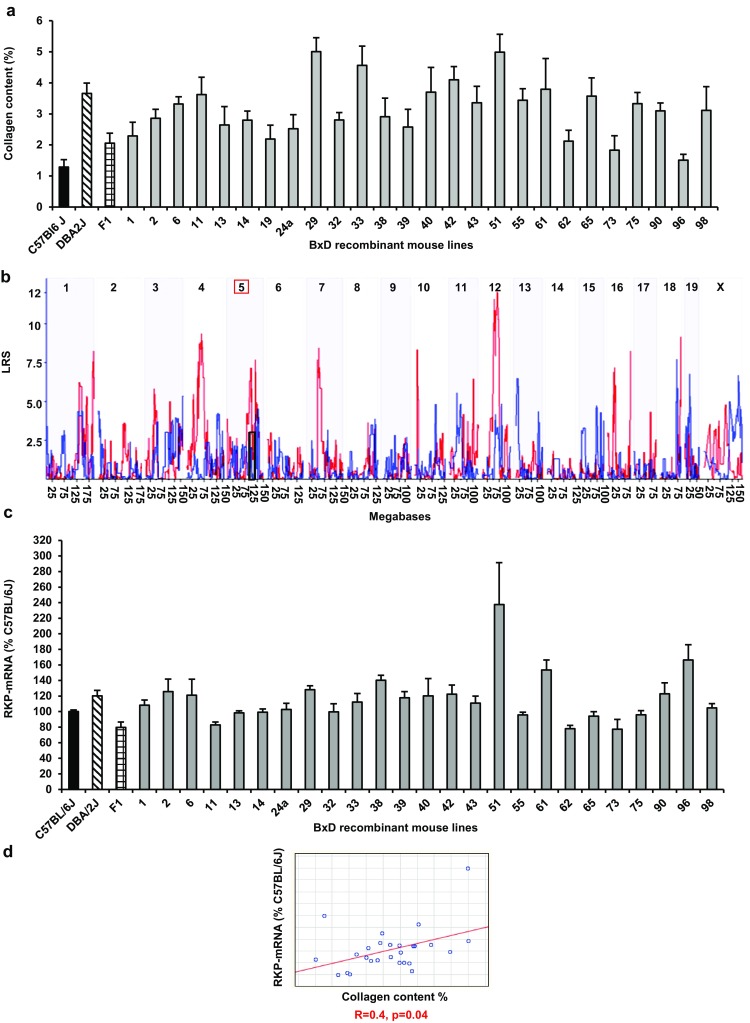
The extent of cardiac fibrosis and QTL analyses for cardiac and hepatic fibrosis in CCl_4_-treated recombinant inbred BxD mouse lines. **a** The extent of cardiac fibrosis, measured by picrosirius red staining, in CCl_4_-treated recombinant inbred lines descending from C57BL/6 J and DBA2/J strains (BxD) (*n* = 10–12 per group). **b** QTL analyses for cardiac (blue line) and hepatic (red line) fibrosis in the BxD reference population. Most QTL are organ-specific but few affect fibrosis across tissues. In particular on chromosome 5, the overlapping susceptibility loci indicated by the black box harbor the *PEBP*-*I* gene locus coding for RKIP. LRS-likelihood ratio statistics. **c** mRNA-expression of RKIP in the LV myocardium of CCl_4_-treated BxD mouse lines (*n* = 3–6 per group). **d** mean values of cardiac collagen content correlate positively with mean values of *RKIP*- mRNA in LV myocardium of BxD mouse lines (*r* = 0.4, *p* = 0.04, B x D *n* = 28)

The genetically mosaic CCl_4_-treated BXD inbred mouse lines displayed significant variation of quantitative fibrosis phenotypes, consistent with polygenic inheritance of fibrosis **(**Fig. 1a**)** [13]. Cardiac fibrosis was quantified morphometrically as fractional area of collagen content using picrosirius red staining. QTL analyses identified *Raf Kinase Inhibitor Protein* (*RKIP*) as potential genetic marker of fibrosis both in the heart and in the liver **(**Fig. 1b, c) [13]. Collagen content in the LV myocardium of the parental strains, the F1 hybrids and recombinant inbred BxD mouse lines correlated positively with the myocardial mRNA-expression of *RKIP* (*r* = 0.4, *p* = 0.04) (Fig. 1c, d). The increased cardiac fibrosis in CCl_4_-treated DBA/2 J was associated with the enhanced myocardial mRNA-expression of *RKIP* (mRNA *RKIP* CCl_4_ DBA/2 J 120 ± 7% vs.CCl_4_ C57BL/6 J 100 ± 2%, *p* = 0.02) and increased ROS production in cardiac fibroblasts (32 ± 7% 8-dOHG^+^ fibroblasts in CCl_4_ DBA/2 J vs. 5 ± 2%, in CCl_4_ C57BL/6 J, *p* = 0.01) **(**Fig. 1a, c).

### Reduced interstitial and replacement fibrosis in CCl_4_-treated and pressure-overloaded *RKIP*^−*/*−^ N mice

**Fig. 2:**
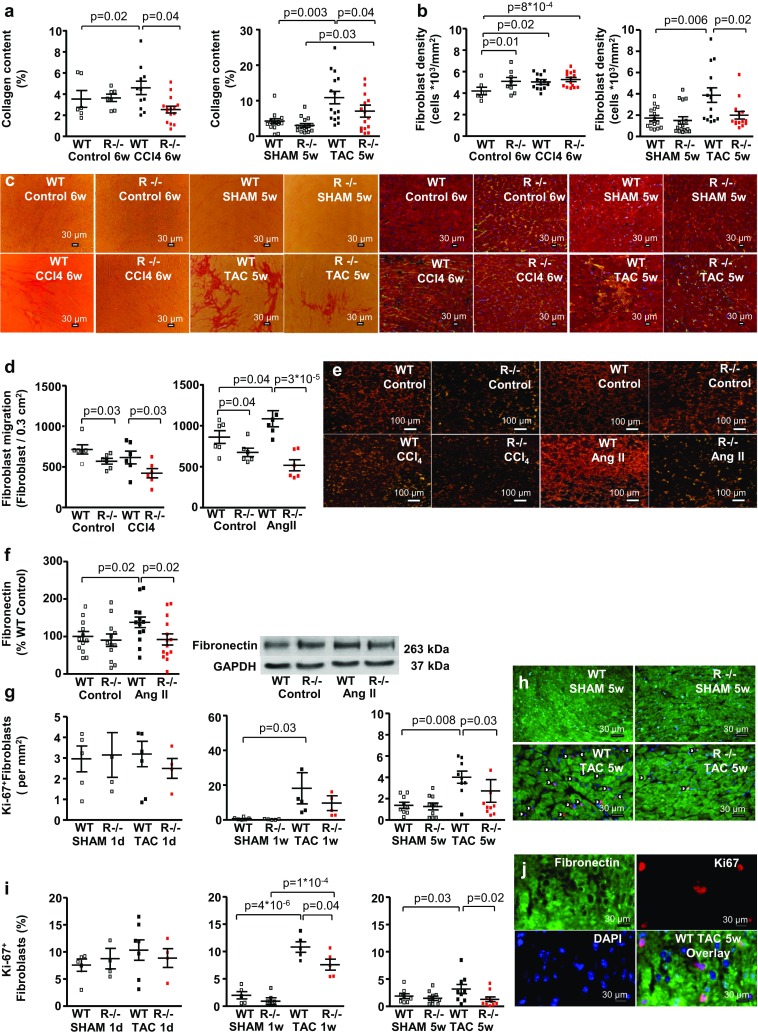
Systemic *RKIP*-deficiency reduces interstitial and replacement cardiac fibrosis, diminishes fibroblast proliferation, migration and production of intracellular fibronectin by cardiac fibroblasts in *RKIP*^−*/*−^ N TAC mice. **a** Systemic *RKIP*-deficiency reduces cardiac fibrosis in CCl_4_-treated and TAC mice (*n* = 6–15 per group), **b** prevents an increase in fibroblast density in CCl_4_- treated N mice and reduces their number in N TAC mice (*n* = 6–15 per group). **c** Representative sections of the LV myocardium stained with picrosirius red and co-immunostaining for myocytic α-sarcomeric actin (red) and the fibroblast marker intracellular fibronectin (green). Nuclei are stained blue by DAPI. Bars = 30 µm. **d** *RKIP*-knockout in adult murine cardiac fibroblast reduces the number of migrating fibroblasts in a Boyden chamber with an insert size of 0.3 cm^2^ under basal conditions and after treatment with CCl_4_ and AngII. AngII but not CCl_4_ increased fibroblast migration. **e** shows representative migration assays. Migrating fibroblasts are immunostained for intracellular fibronectin (red). Bars = 100 µm. **f** *RKIP*-knockout in adult murine cardiac fibroblasts reduces AngII-stimulated protein expression of intracellular fibronectin (*n* = 7–11 per group). **g**, **i** Pressure overload in N mice for 1 week (*n* = 5–6 per group) and 5 weeks, significantly increases **g** the number per mm^2^ and **i** the percentage of cycling Ki67^+^fibronectin^+^ fibroblasts in the LV myocardium which were further reduced by *RKIP*-knockout (*n* = 9–10 per group). Acute pressure overload did not influence both parameters (*n* = 4–8 per group). **h** Representative sections of the LV myocardium stained with co-immunostaining for Ki67^+^ (red) and myocytic α-sarcomeric actin (green). Cycling nuclei are marked by arrowheads. **j** Ki67^+^ fibroblasts in a replacement fibrosis in LV section from a WT N TAC mouse: intracellular fibronectin (green), Ki67 (red), nuclei stained blue by DAPI and the overlay of the three stainings. Bars = 30 µm. Mann–Whitney-test, one-way ANOVA with Fisher LSD post hoc test

To induce interstitial cardiac fibrosis, mice were treated with carbon tetrachloride (CCl_4_), an established inductor of systemic fibrosis. CCl_4_ treatment for 6 weeks did not elicit cardiac hypertrophy and did not increase peripheral blood pressure in WT and *RKIP*^−*/*−^ animals (Table 1). Cardiac fibrosis was quantified morphometrically as fractional area of collagen content using picrosirius red staining. Systemic RKIP-knockout diminished CCl_4_-induced interstitial fibrosis (Fig. 2a, c), prevented an increase of fibroblast density (Fig. 2b, c) and reduced myocardial *collagen Iα2 (COL1A2)* mRNA expression (Suppl. Figure 1a).

**Table 1:** Effects of carbon tetrachloride (CCl_4_) and RKIP^−/−^ on parameters of myocardial hypertrophy

	WT C57BL/6 N control	RKIP^−/−^ C57BL/6 N control	WT C57BL/6 N CCl_4_	RKIP^−/−^C57BL/6 N CCl_4_
Animal number	6	8	14	16
Body weight (g)	23 ± 1.6	25 ± 0.6	26 ± 1.1	25 ± 1.2
Heart rate (bpm)	550 ± 9	478 ± 6	505 ± 7	510 ± 7
SABP (mmHg)	111 ± 0.8	106 ± 0.9	112 ± 1.1	110 ± 0.9
DABP (mmHg)	82 ± 1.5	81 ± 1.2	83 ± 1.6	81.5 ± 1.2
Lung fluid weight/TL (mg/mm)	9. ± 0.8	9 ± 0.6	11 ± 0.5*	11 ± 0.6*
Heart weight/TL (mg/mm)	7.5 ± 0.5	8.6 ± 0.8	8 ± 0.4	8 ± 0.3
CCSA (μm^2^)	256 ± 20	272 ± 14	255 ± 18	252 ± 14
Cardiomyocytes/mm^2^	4519 ± 369	4159 ± 179	4400 ± 135	4593 ± 276

Data are presented as mean values ± SEM. One-way ANOVA with a Fisher LSD post hoc test, *p < 0.05 vs. corresponding control group

*CCSA* cardiomyocyte cross-sectional area, *LV* left ventricle, *LVEDP* left ventricular end-diastolic pressure, *LVSP* left ventricular systolic pressure, *TL* tibia length

Replacement cardiac fibrosis was elicited by chronic cardiac afterload induced by TAC. 5 weeks post TAC left ventricular systolic pressure (LVSP) and left ventricular end-diastolic pressure (LVEDP) were increased as expected (Table 2). TAC induced myocardial and cardiomyocyte hypertrophy both of which were reduced in *RKIP*^−*/*−^ mice (Table 2). Systemic RKIP-deficiency decreased TAC-induced replacement fibrosis (Fig. 2a, c), diminished fibroblast density (Fig. 2b, c), reduced myocardial *collagen Iα2 (COL1A2)* and *connective tissue growth factor (CTGF)* mRNA expression (Suppl. Figure 1a, b) in *RKIP*^−*/*−^ N TAC. Isolated adult cardiac fibroblasts (ACF) of *RKIP*^−*/*−^ N mice demonstrated reduced migration capacity in a modified Boyden chamber, untreated and after treatment with CCl_4_ or angiotensin II (Ang II) (Fig. 2d, e). ACF of *RKIP*^−*/*−^ N mice did not increase the production of intracellular fibronectin in cell culture after treatment with Ang II (Fig. 2f).

**Table 2:** Effects of TAC and RKIP^−/−^ on parameters of myocardial hypertrophy

	WT C57BL/6 N SHAM	RKIP^−/−^C57BL/6 N SHAM	WT C57BL/6 N TAC	RKIP^−/−^C57BL/6 N TAC
Animal number	15	16	16	16
Body weight (g)	28 ± 1.3	25 ± 1.0	25.5 ± 1.0	25 ± 0.5
Heart rate (bpm)	211 ± 15	185 ± 14	198 ± 15	202 ± 17
LVSP (mmHg)	92 ± 7	83 ± 6	122 ± 6*	112 ± 5*
LVdP/d*t*_max_ (mmHg/s)	3214 ± 67	2723 ± 228	2948 ± 136	3090 ± 211
LVdP/d*t*_min_ (mmHg/s)	− 2963 ± 67	− 2567 ± 266	− 3108 ± 205	− 3075 ± 240
LVEDP (mmHg)	18 ± 4	18 ± 3	40 ± 6*	30 ± 4*†
Lung fluid weight/TL (mg/mm)	10 ± 0.3	9.7 ± 0.5	12 ± 0.9*	10 ± 0.5
Heart weight/TL (mg/mm)	8.7 ± 0.5	8.7 ± 0.4	13 ± 1.5*	10 ± 0.8*†
CCSA (μm^2^)	302 ± 13	272 ± 20‡	478 ± 18*	299 ± 23*†
Cardiomyocytes/mm^2^	3317 ± 191	4192 ± 212‡	2310 ± 313*	3617 ± 205*†

Data are presented as mean values ± SEM. One-way ANOVA with a Fisher LSD post hoc test, *p < 0.05 vs. corresponding control group; †*p* < 0.05 vs. WT C57BL/6 N TAC; ‡*p* < 0.05 vs. WT C57BL/6 N SHAM

*CCSA* cardiomyocyte cross-sectional area, *LV* left ventricle, *LVEDP* left ventricular end-diastolic pressure, *LVSP* left ventricular systolic pressure, *TL* tibia length

### Reduced number of proliferating and CXCR4^+^fibroblasts and diminished oxidative stress in pressure-overloaded myocardium of *RKIP*^−*/*−^ N TAC mice

The percentage of cycling Ki67^+^ fibroblasts in the LV myocardium was diminished in *RKIP*^−*/*−^ N mice 1 week and 5 weeks post TAC, compared with corresponding WT N TAC **(**Fig. 2g–j).

Both CCl_4_ treatment and TAC increased apoptosis in cardiomyocytes, non-cardiomyocyte cells and fibroblasts (Suppl. Figure 2, 3) accompanied by increased number of cycling cardiomyocytes and cardiac fibroblasts identified by expression of Ki67 (Suppl. Figure 4). Systemic *RKIP*-deficiency ameliorated cardiac cell turnover in both models (Suppl. Figure 2–4).

**Fig. 3:**
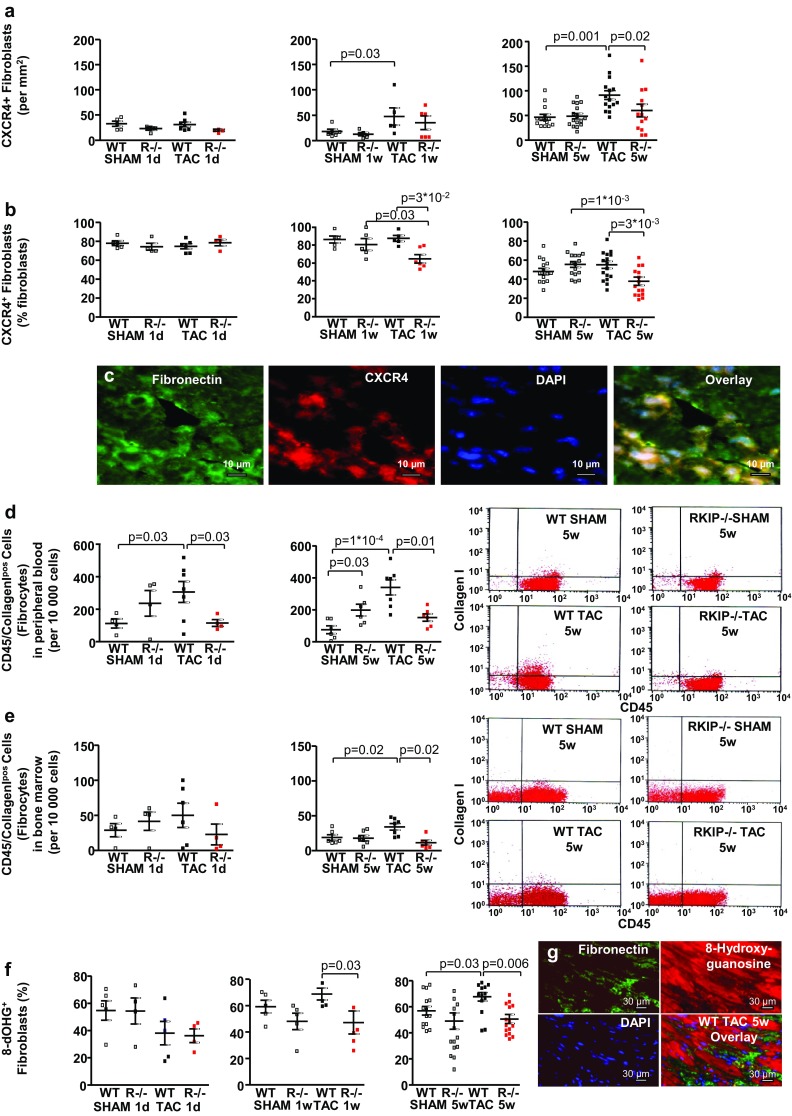
Systemic *RKIP*-deficiency reduces the number of CXCR^+^ fibroblasts and circulating fibrocytes in the time course of cardiac fibrogenesis and ameliorates myocardial oxidative stress in *RKIP*^−*/*−^ N TAC mice. **a**, **b** *RKIP*^−*/*−^ N TAC mice demonstrate reduced number per mm^2^ (**a**) and reduced percentage (**b**) of CXCR4^+^ fibroblasts in the LV myocardium 1 week (*n* = 5–6 per group) and 5 weeks post TAC (*n* = 14–16 per group). Acute pressure overload does not influence both parameters (*n* = 4–8 per group). **c** CXCR4^+^ fibroblasts in a LV section from a WT N TAC mouse: intracellular fibronectin (green), CXCR4 (red), nuclei stained blue by DAPI and the overlay of the three stainings. Bars = 10 µm. **d**, **e** The numbers of CD45/collagen I-positive fibrocytes quantified by FACS were increased in the peripheral blood 1 day post TAC (*n* = 4–7 per group) and in the peripheral blood (**d**) and BM (*n* = 6–7 per group) (**e**) 5 weeks post TAC and was significantly reduced by *RKIP*-knockout. **d**, **e** show representative FACS blots for CD45 (*X*-axis) and collagen I (*Y*-axis) in the peripheral blood and in the BM respectively 5 weeks after surgery. The double positive cells in the upper right quadrant were counted as fibrocytes. One-way ANOVA with Fisher LSD post hoc test. **f** The myocardial oxidative stress was evaluated as the percentage of 8-dOHG^+^fibronectin^+^ fibroblasts which was significantly increased 1 week (*n* = 5–6 per group) and 5 weeks (*n* = 14–16 per group) post TAC and significantly reduced in *RKIP*^−*/*−^ N TAC mice. Acute pressure overload does not influence the percentage of 8-dOHG^+^ fibronectin^+^ fibroblasts (*n* = 4–8 per group). **g** Co-immunostaining for intracellular fibronectin and 8-dOHG in a LV section from a WT N TAC mouse: intracellular fibronectin (green), 8-dOHG (red), nuclei stained blue by DAPI and the overlay of the three stainings. Bars = 30 µm

The SDF-1/CXCR4 axis plays pivotal role in the regulation of fibroblast activity driving proliferation, migration and collagen production as well as in the mobilisation of circulating fibroblasts from the bone marrow and their recruitment into the myocardium [16, 17]. Co-immunostaining for intracellular fibronectin and CXCR4 was used for the histological evaluation of the influence of TAC and *RKIP*-knockout on myocardial fibroblasts. The percentage of CXCR4^+^ fibroblasts was reduced in *RKIP*^−*/*−^ N mice 1 week and 5 weeks post TAC **(**Fig. 3a–c**)**. Systemic *RKIP*-deficiency significantly reduced the number of CD45^+^ collagen I^+^ fibrocytes in the peripheral blood during acute and chronic pressure overload **(**Fig. 3d). TAC for 5 weeks significantly increased the number of fibrocytes in the BM which was reduced in *RKIP*-knockout (Fig. 3e).

Systemic *RKIP*-deficiency significantly reduced oxidative stress elicited by chronic pressure overload in *RKIP*^−*/*−^ N TAC mice evaluated by co-immunostaining for 8-dOHG and intracellular fibronectin (Fig. 3f, g).

### Differential effects of acute and chronic pressure overload and systemic RKIP-deficiency on nuclear expression of Nrf2 in the myocardium

**Fig. 4:**
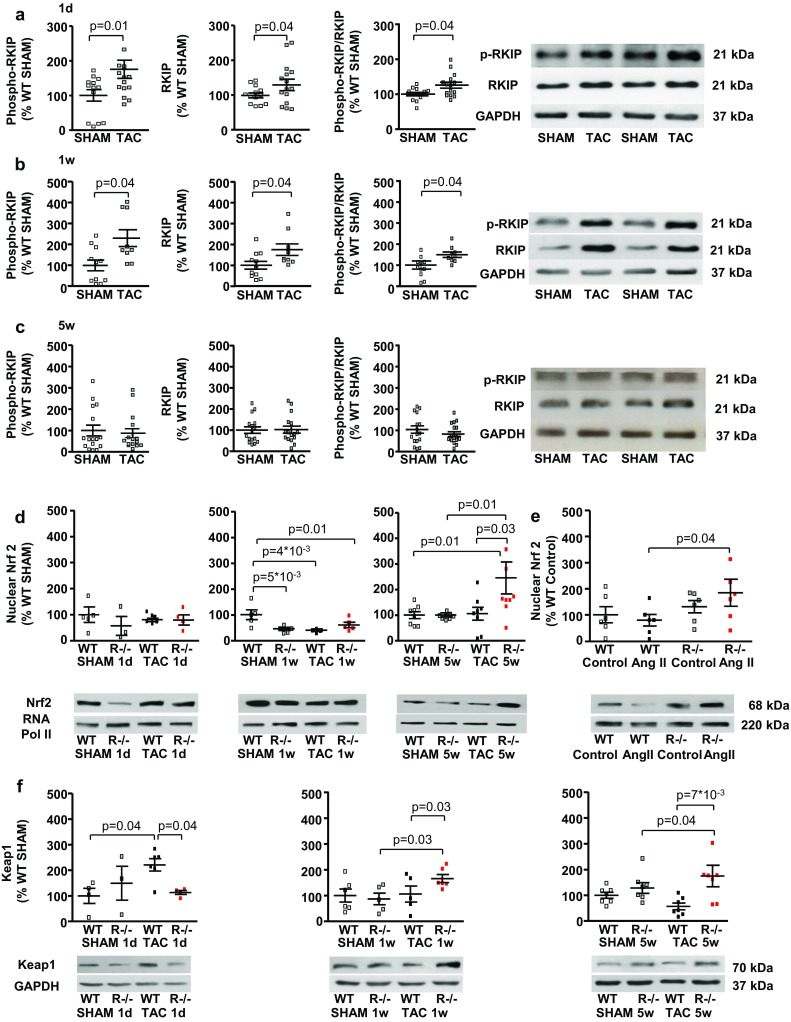
Nuclear protein accumulation of Nrf2 is differently regulated by RKIP and enhanced by its knockout in the time course of pressure overload in N TAC mice. **a**, **b** Both the whole protein expression and phosphorylation of RKIP (Ser 153) were increased 1 day (**a**) (*n* = 13–15 per group) and 1 week (**b**) (*n* = 9–11 per group) post TAC in N mice. **c** There was no significant difference in protein expression and phosphorylation of RKIP (Ser 153) 5 week post TAC (*n* = 16 per group). **d** The nuclear protein accumulation of Nrf2 was not changed by 1 day-TAC (*n* = 3–6 per group) but was significantly reduced 1 week (*n* = 4–5 per group) post TAC in WT N mice. Systemic *RKIP*-deficiency slightly increased the nuclear protein accumulation of Nrf2 1 week post TAC and drastically increased it 5 weeks post TAC. **e** *RKIP*-knockout increases nuclear protein accumulation of Nrf2 in adult murine cardiac fibroblasts treated with Ang II (*n* = 6 per group). **f** Acute pressure overload significantly increased protein expression of Keap1 (*n* = 3–6 per group), which, however, was reduced 1 week (*n* = 5–6 per group) and 5 weeks (*n* = 7 per group) post TAC. *RKIP*-knockout enhanced protein expression of Keap1 1 week and 5 weeks post TAC. Mann–Whitney-test, one-way ANOVA with Fisher LSD post hoc test

At the first day and 1 week after TAC protein expression and phosphorylation of RKIP were increased compared with SHAM, while this difference vanished after 5 weeks **(**Fig. 4a–c). Both *RKIP*-knockout and TAC increased phosphorylation of extracellular signal-regulated kinase 1/2 (ERK1/2) which remained unchanged during the first week after TAC surgery (Suppl. Figure 5a, b) but was significantly reduced in C57BL/6 N 5 weeks post TAC (Suppl. Figure 5c).

The nuclear localization of the main transcriptional activator of antioxidant proteins, nuclear factor erythroid 2-related factor 2 (Nrf2), was significantly reduced during the first week of TAC (Fig. 4d). Systemic *RKIP*-deficiency drastically increased the nuclear accumulation of Nrf2 accompanied by an enhanced protein expression of its negative regulator, Kelch-like ECH-associated protein 1 (Keap1), during chronic pressure overload **(**Fig. 4d, f).

Furthermore, cultured adult cardiac fibroblasts from *RKIP*^−*/*−^ N mice treated with Ang II demonstrated increased nuclear accumulation of Nrf2 (Fig. 4e). Co-immunostainings for Nrf2 and myocytic α-sarcomeric actin and intracellular fibronectin revealed an increased nuclear localization of Nrf2 in pressure-overloaded cardiomyocytes and fibroblasts of RKIP^−/−^ N mice (Suppl. Figure 6a, b).

### Reduced Jak2/Fyn myocardial signaling in RKIP^−*/*−^ N mice

**Fig. 5:**
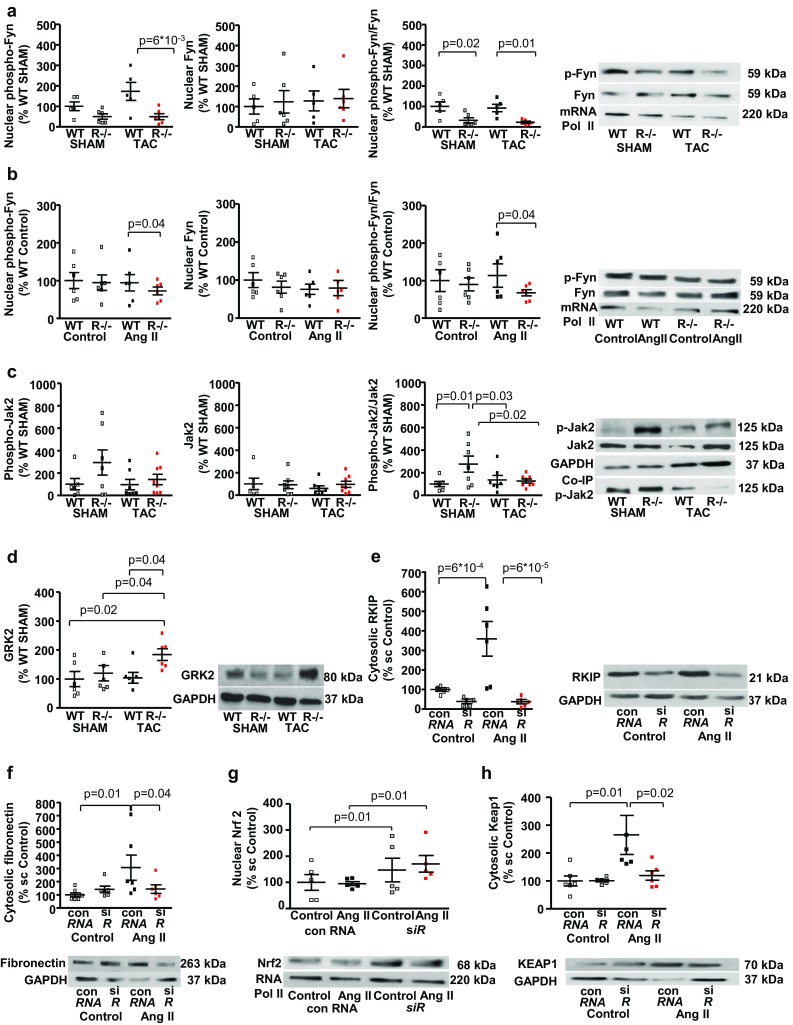
Systemic *RKIP*-deficiency down-regulates Jak2/Fyn myocardial signaling in *RKIP*^−*/*−^ N TAC mice. **a** Systemic *RKIP*-deficiency significantly reduced nuclear expression of the active form of Thr12 p-Fyn in both groups 5 weeks after surgery (*n* = 5–6 per group). **b** *RKIP*-knockout reduces nuclear expression of p-Fyn in adult murine cardiac fibroblasts treated with AngII (*n* = 6 per group). **c** Systemic *RKIP*-deficiency significantly increased myocardial expression of the active form of Tyr1007/1008 p-Jak2 in *RKIP*^−*/*−^ SHAM, which was drastically reduced after 5 weeks of TAC (*n* = 6–8 per group). Heart tissue from WT and *RKIP*^−*/*−^ was immunoprecipitated with anti-Jak2 antibody 5 weeks after surgery and then western blot for Tyr1007/1008 p-Jak2 was performed. *RKIP*^−*/*−^TAC N demonstrates diminished phosphorylation of Jak2. **d** Systemic *RKIP*-deficiency significantly enhanced myocardial expression of GRK2 after 5 weeks of TAC (*n* = 6 per group). Acute reduction of *RKIP* expression (**e**) in adult cardiac fibroblasts of C57BL/6N with siRNA (siR) significantly diminished the AngII-induced cytosolic protein expression of fibronectin (**f**) and increased the nuclear accumulation of Nrf2 (**g**) in cells treated with scrambled control (sc) RNA (con RNA) and AngII. AngII increased the cytosolic protein expression of RKIP (**e**) and Keap1 (**h**) in ACF (*n* = 5–6 per group). One-way ANOVA with Fisher LSD post hoc test

The non-receptor protein tyrosine kinase Fyn phosphorylates Nrf2 inducing its export from the nucleus and proteasomal degradation [36]. Systemic *RKIP*-deficiency significantly reduced the nuclear accumulation of the active phosphorylated form Thr12 phospho-Fyn in both groups 5 weeks after surgery and in cultured adult cardiac fibroblasts treated with Ang II (Fig. 5a, b).

In response to Ang II, Janus kinase 2 (Jak 2) activation is required for Fyn activation [37]. Both Western blot analyses of Jak2 protein and co-immunoprecipitation with anti-Jak2 antibody demonstrated an increased myocardial expression of the active form of Tyr1007/1008 phospho-Jak2 in *RKIP*^−*/*−^ N SHAM mice, which was significantly reduced after 5 weeks of TAC (Fig. 5c).

Phospho-RKIP inhibits G-protein coupled receptor kinase 2 (GRK2), which subsequently induces desensitization and internalization of angiotensin II type 1 (AT1)-receptors [3, 34]. Systemic *RKIP*-deficiency significantly enhanced myocardial expression of GRK2 after 5 weeks of pressure overload (Fig. 5d). To further confirm that the observed effects are caused by RKIP-inhibition in cardiac fibroblasts, ACF of C57BL/6N mice were treated with siRNA for *RKIP* (siR) and AngII. Untreated ACF and scrambled control (sc) RNA (con RNA) were used for control experiments (*n* = 5–6 per group) (Fig. 5e–h). The treatment with Ang II significantly increased protein expression of RKIP, intracellular fibronectin, and Keap1 (Fig. 5e, f, h). si*RKIP* markedly reduced both basal and AngII-stimulated protein expression of RKIP accompanied by the increased nuclear accumulation of Nrf2 in both groups and a reduction of intracellular fibronectin in AngII-treated fibroblasts (Fig. 5e–g).

### Differential effects in *RKIP*^−*/*−^N- versus *RKIP*^−*/*−^J mice after pressure overload

**Fig. 6:**
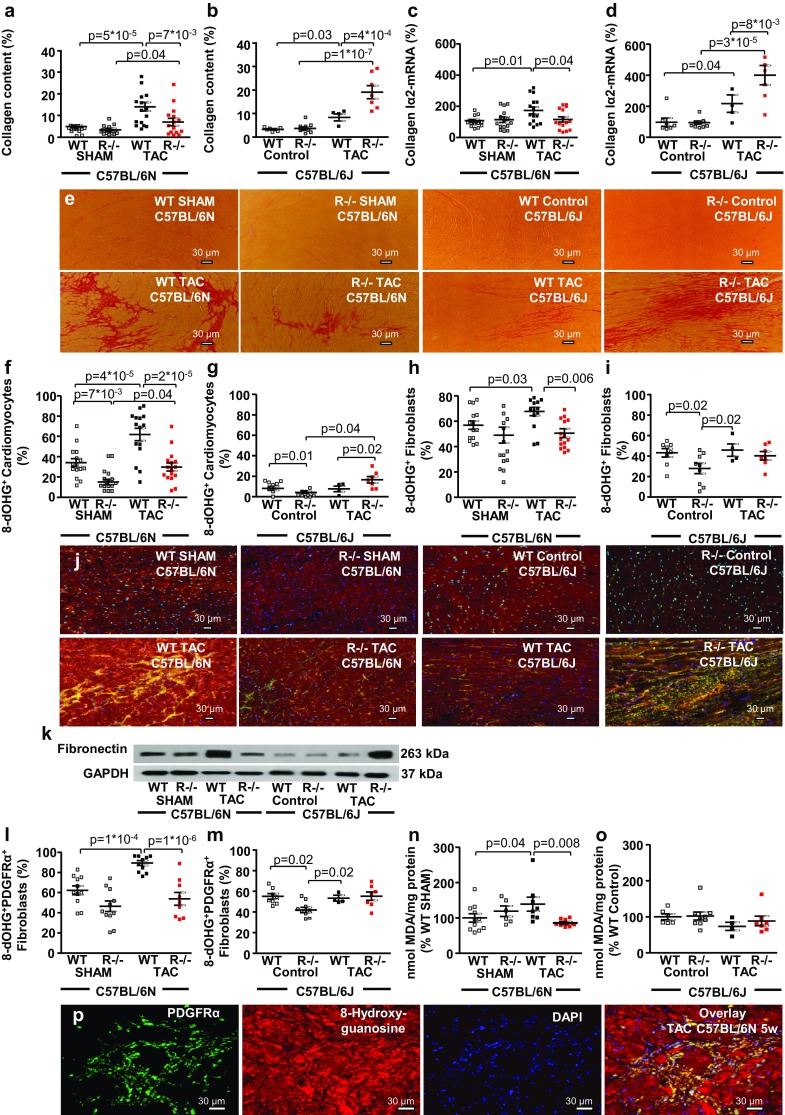
Amelioration of cardiac fibrosis in *RKIP*^−*/*−^N versus *RKIP*^−*/*−^J mice after pressure overload. **a**, **b** Systemic *RKIP* deficiency (**a**) reduces cardiac fibrosis in *RKIP*^−*/*−^ N TAC (*n* = 15 per group) (**b**) but increases in *RKIP*^−*/*−^J TAC mice (*n* = 5–9 per group). **c** Systemic *RKIP*-deficiency diminishes mRNA-expression of *collagen Ia2* in *RKIP*^−*/*−^N TAC (*n* = 15 per group), **d** but enhances in *RKIP*^−*/*−^J TAC mice (*n* = 4–9 per group). **e** Representative sections of the LV myocardium stained with picrosirius red. Bars = 30 µm. Myocardial oxidative stress in cardiomyocytes and fibroblasts was evaluated by co-immunostaining for 8-dOHG and α–sarcomeric actin and intracellular fibronectin respectively. **f** Myocardial oxidative stress was significantly reduced in *RKIP*^−*/*−^N SHAM and *RKIP*^−*/*−^N TAC compared with control groups (*n* = 15–16 per group). **g** The percentage of 8-dOHG^+^ cardiomyocytes was significantly reduced in *RKIP*^−*/*−^J Control and was slightly increased in *RKIP*^−*/*−^J TAC compared with control groups (*n* = 4–9 per group). **h** The percentage of 8-dOHG^+^ fibroblasts in N mice was increased by TAC and diminished by *RKIP*-knockout (*n* = 15–16 per group). **i** *RKIP*^−*/*−^J Control mice demonstrated reduced basal ROS production in fibroblasts which was not further changed by TAC (*n* = 4–9 per group). **j** Representative sections of the LV myocardium with co-immunostaining for the 8-dOHG (red) and intracellular fibronectin (green). Nuclei are stained blue by DAPI. **k** Representative western blot demonstrating different expression of the fibroblast marker intracellular fibronectin in WT and *RKIP*^−*/*−^ TAC in N and J strains. **l**, **m** Co-immunostaining for 8-dOHG and fibroblast marker PDGFRα further confirms the differential effects of *RKIP*-knockout on oxidative stress and fibrosis in N (*n* = 9–11 per group) and J (*n* = 4–9 per group) strains. **n**, **o** The myocardial lipid peroxidation was evaluated by measuring the concentration of malondialdehyde (MDA) in the left ventricular myocardium. TAC during 5 weeks significantly increased the myocardial concentration of MDA in WT N TAC but not in WT J TAC mice. Systemic *RKIP*-deficiency reduced the MDA concentration in LV myocardium of *RKIP*^−*/*−^N TAC (*n* = 6-11 per group) and did not change it in *RKIP*^−*/*−^J TAC mice (*n* = 4–9 per group). **p** Co-immunostaining for PDGFRα and 8-dOHG in a LV section from a WT N TAC mouse: PDGFRα (green), 8-dOHG (red), nuclei stained blue by DAPI and the overlay of the three stainings. Bars = 30 µm. One-way ANOVA with Fisher LSD post hoc test

To further assess the role of oxidative stress for the observed effects of RKIP, C57BL/6 N and C57BL/6 J mice were compared. We have previously demonstrated that the nicotinamide nucleotide transhydrogenase (Nnt) plays a very important role for the ROS-production and enhanced cardiac fibrosis in the pressure-overloaded myocardium [29]. Due to the mutation of the *Nnt* gene, the inbred mouse strain C57BL/6 J is protected from oxidative stress and fibrosis in response to pressure overload [29]. Indeed, systemic *RKIP* deficiency caused differential effects on cardiac fibrogenesis in pressure-overloaded myocardium of the two mouse strains: cardiac fibrosis and *COL1A2* mRNA expression were reduced in *RKIP*^−*/*−^ N after TAC but increased in *RKIP*^−*/*−^ J TAC compared with the corresponding wild types **(**Fig. 6a–e).

Oxidative stress in cardiomyocytes and cardiac fibroblasts was evaluated by co-immunostaining for 8-dOHG and α–sarcomeric actin and intracellular fibronectin respectively. TAC significantly increased ROS production both in cardiomyocytes and fibroblasts of WT N but not WT J mice (Fig. 6f–j). *RKIP*^−*/*−^N mice demonstrated decreased percentage of 8-dOHG^+^ cardiomyocytes in both groups (Fig. 6f). In contrast, the percentage of 8-dOHG^+^ cardiomyocytes in *RKIP*^−*/*−^J mice was lower in the control group but was significantly enhanced by TAC (Fig. 6g). The percentage of 8-dOHG^+^ fibroblasts was diminished by RKIP-knockout and not affected by TAC in both mouse strains (Fig. 6h, i). Representative western blot demonstrates different expression of intracellular fibronectin in the experimental groups (Fig. 6k).The observations were further confirmed by the co-immunostaining for 8-dOHG and the fibroblast marker platelet-derived growth factor receptor alpha (PDGFRα) (Fig. 6l, m, p). Pressure overload during 5 weeks significantly increased the myocardial concentration of malondialdehyde (MDA) in WT N TAC but not in WT J TAC **(**Fig. 6n, o). Systemic RKIP-deficiency reduced MDA concentration in *RKIP*^−*/*−^ N TAC and did not change it in *RKIP*^−*/*−^ J TAC (Fig. 6n, o).

**Fig. 7:**
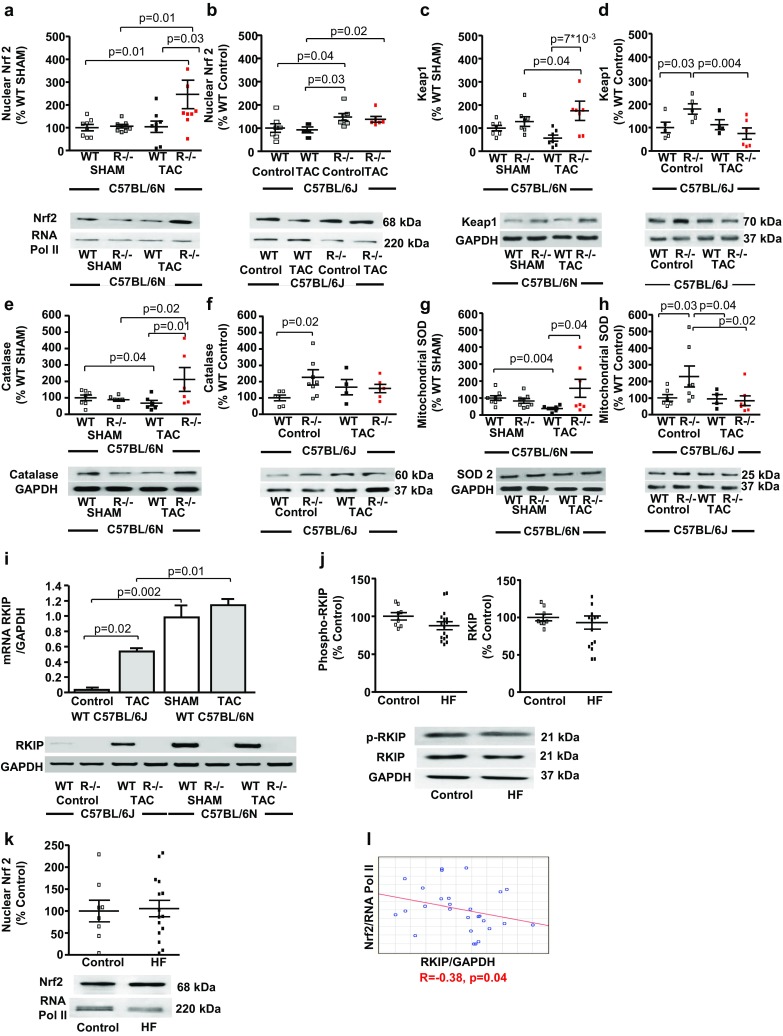
Expression of RKIP and Nrf2 in the LV of N and J mice and myocardial samples from human non-failing and failing LV. **a** *RKIP*^−*/*−^ N TAC (*n* = 8 per group) showed a twofold increase of nuclear accumulation of Nrf2 and protein expression of Keap1 (**c**). **b** *RKIP*^−*/*−^ J Control mice (*n* = 4–7 per group) revealed enhanced the basal nuclear accumulation of Nrf2 and the protein expression of Keap1 (**d**), which was not further enhanced by TAC. **e**, **f** The enhanced nuclear accumulation of Nrf2 in *RKIP*^−*/*−^ N TAC (**e**) and *RKIP*^−*/*−^ J Control (**f**) was accompanied by an increased myocardial protein expression of the Nrf2-regulated antioxidative enzyme catalase and mitochondrial superoxide dismutase/SOD2 (**g**, **h**). **i** Both basal and TAC-induced mRNA expression of the gene coding for RKIP, *phosphatidylethanolamine binding protein I* (*PEBP*-*I*), was significantly lower in J than in N mice but was increased by TAC in J mice. The protein expression of p-RKIP, RKIP (**j**) and nuclear *RKIP*^−*/*−^N TAC (**k**) was similar in myocardial samples from human non-failing (*n* = 8) and failing (*n* = 15) LV myocardium. **l** Protein expression of RKIP correlated negatively with the nuclear expression of Nrf2 (*r* = − 0.38, *p* = 0.04). Mann–Whitney-test, one-way ANOVA with Fisher LSD post hoc test, Spearman correlation analysis

Nuclear accumulation of Nrf2 and protein expression of Keap1 were differentially regulated by *RKIP*-knockout in the two mouse strains. *RKIP*^−*/*−^ N TAC mice revealed a two-fold increased nuclear accumulation of Nrf2 and protein expression of Keap1, compared with WT N TAC (Fig. 7a, c). In contrast, *RKIP*^−*/*−^ J Control mice demonstrated increased basal nuclear accumulation of Nrf2 and protein expression of Keap1 that were not further enhanced by TAC (Fig. 7b, d). The increased nuclear accumulation of Nrf2 was accompanied by enhanced protein expression of catalase and mitochondrial superoxide dismutase in *RKIP*^−*/*−^N TAC and *RKIP*^−*/*−^ J Control mice (Fig. 7e–h).

**Fig. 8:**
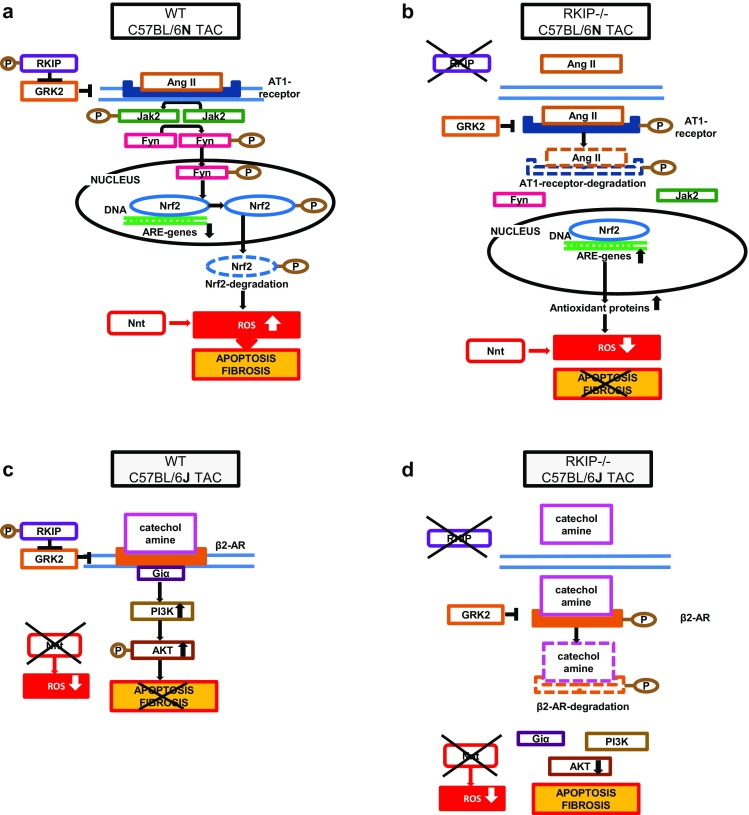
Schematic depiction of the results and proposed hypothesis. **a** In pressure-overloaded left-ventricular myocardium of WT N TAC mice Nnt is the dominant source of ROS and phospho-RKIP inhibits GRK2, preventing the down-regulation of AT1-receptor. TAC-induced production of AngII activates the AT1-Jak2-Fyn signaling cascade, leading to phosphorylation of Nrf2, its rapid translocation from the nucleus and degradation by proteasomes [36, 37]. The decreased nuclear content of Nrf2 diminishes the expression of antioxidant, cytoprotective ARE-genes. The enhanced myocardial ROS production leads to cell death and fibrosis. **b** Systemic *RKIP*-knockout in *RKIP*^−*/*−^N TAC leads to the activation of GRK2, which phosphorylates AT1-receptor, eliciting its internalization and degradation [34]. The downregulation of AT1-Jak2-Fyn signaling cascade inhibits nuclear export of Nrf2 [36]. The nuclear accumulation of Nrf2 enhances the expression of ARE-genes reducing myocardial ROS production, cardiac apoptosis, and fibrosis. **c** Due to the mutation of the *Nnt* gene, the inbred mouse strain C57BL/6 J is protected from oxidative stress and fibrosis in response to pressure overload [24]. In the pressure-overloaded myocardium of WT J TAC mice phospho-RKIP inhibits GRK2, preventing β2-adrenergic receptor (AR) repression. The catecholamines stimulate β2-AR, activating the protective β2-AR-Giα-PI3 K-AKT signaling pathway, thus preventing cardiac apoptosis and fibrosis [38, 40]. **d** Systemic *RKIP*-knockout in *RKIP*^−*/*−^J TAC mice enhances the activity of GRK2 that desensitizes β2-AR through phosphorylation at Ser 355/356, diminishing the activation of β2-AR-Giα-PI3 K-AKT signaling pathway [10, 38]. The reduced activation of the protective β2-Giα-PI3 K-AKT signaling pathway increases cardiac apoptosis and fibrosis despite the reduced oxidative stress in *RKIP*^−*/*−^J TAC mice

The expression of the gene coding for RKIP, *PEBP*-*I*, assessed by RT-PCR was differentially influenced by TAC in the two mouse strains: *PEBP*-*I* was increased in J and was not changed in N (Fig. 7i). Furthermore, both basal and TAC-induced expression of *PEBP*-*I* was significantly lower in J than in N mice (Fig. 7i). The differential effects of *RKIP*-knockout in pressure-overloaded N and J mice are summarized in Fig. 8 [10, 38].

### Negative correlation of RKIP protein expression with the nuclear accumulation of Nrf2 in myocardial samples from human non-failing and failing hearts

The RKIP protein expression correlated negatively with the nuclear accumulation of Nrf2 in myocardial samples from human non-failing and failing left ventricles (*r* = − 0.38, *p* = 0.04). There were no differences in protein expression of phospho-RKIP, RKIP and nuclear protein content of Nrf2 **(**Fig. 7j–l).

## Discussion

The study identifies the importance of the Raf Kinase Inhibitor Protein (RKIP) for cardiac fibrogenesis. Importantly, we observed a differential regulation of cardiac fibrogenesis by RKIP depending on the myocardial redox state. C57BL/6-*RKIP*-deficient (*RKIP*^−*/*−^) N mice subjected to increased afterload (TAC) exhibit reduced myocardial fibrosis and oxidative stress. The mechanism relates to upregulation of the nuclear factor erythroid 2-related factor 2 (Nrf2), the main transcriptional activator of antioxidant proteins. In contrast, the *Nnt*-deficient *RKIP*^−*/*−^ J TAC mice reveal diminished oxidative stress, increased LV fibrosis and enhanced nuclear Nrf2.

We identified RKIP as mediator of cardiac fibrosis associated with increased oxidative stress in the myocardium as result of the unbiased approach using genome wide QTL analyses in BxD lines. During our detailed research of the time course and the associated RKIP signalling, two investigations were published that revealed beneficial as well as detrimental myocardial effects of RKIP in mice [11, 38]. Our data provide the explanation for these potentially discrepant observations showing that the differential effects can be caused by the different genetic backgrounds of the mouse strains and different expression level of RKIP. We have recently reported that C57BL/6 J but not C57BL/6 N mice are protected from myocardial oxidative stress, cardiomyocyte apoptosis and cardiac fibrosis in response to pressure overload due to a mutation of the nicotinamide nucleotide transhydrogenase (Nnt) gene [29]. During cardiac pressure overload, Nnt functions in reverse-mode and, by depletion of antioxidant NADPH, facilitates mitochondrial ROS emission [29]. As a result, profound differences between the N and the J strain with regard to the influence of RKIP on cardiac fibrogenesis are observed.

### Effects of RKIP in C57BL/6-N mice

The analysis of the inbred BxD mouse lines revealed the association of RKIP with increased cardiac fibrosis and ROS production. Therefore we used *RKIP*^−*/*−^ N mice to investigate the influence of RKIP on myocardial fibrosis and oxidative stress. Systemic *RKIP*-deficiency in the Nnt-positive N-strain significantly diminished both CCl_4_- and TAC-induced interstitial and replacement cardiac fibrosis, fibroblast proliferation as well as the expression of fibrotic mediators such as *CTGF* and *collagen*. The activation of SDF-1/CXCR4 axis drastically increases fibroblast activity and migration during cardiac fibrogenesis [16, 17]. Enhanced expression of SDF-1 in inflammatory cardiomyopathy is associated with increased fibrosis and mortality [46]. Systemic *RKIP*-deficiency significantly reduces the number and the percentage of CXCR4^+^ fibroblasts ameliorating LV remodeling during pressure overload. Adult *RKIP*^−*/*−^ N cardiac fibroblasts show reduced migration capacity basal and after treatment with CCl_4_ and angiotensin II, decreased angiotensin II-stimulated production of intracellular fibronectin. In agreement with recent reports in the literature, [3, 11] TAC and CCl_4_-treated RKIP^−/−^ N mice reveal decreased apoptosis and decreased proliferation of cardiomyocytes and non-cardiomyocytes. Thus, systemic *RKIP*-deficiency in C57BL/6 N mice ameliorates interstitial and replacement cardiac fibrosis and cardiac cell turnover.

In addition to the resident cardiac fibroblasts, circulating bone marrow-derived fibroblasts (fibrocytes) contribute to cardiac fibrosis [17, 42, 43]. Although recent experimental findings question the participation of circulating fibrocytes in pressure-overload induced fibrosis, clinical studies demonstrate an association between their elevated number in the peripheral blood and adverse clinical outcomes [1, 19, 27]. Thus, the reduction of the fibrocyte numbers in the peripheral blood and bone marrow of pressure-overloaded C57BL/6 N is consistent with an ameliorative effect of systemic *RKIP*-knockout.

Acute pressure overload increases ERK1/2 phosphorylation in WT which remains unchanged during the first week but is reduced below the basal level 5 weeks post TAC [24]. Basal phosphorylation of ERK1/2 is enhanced by *RKIP*-knockout and not changed by TAC. Recent reports demonstrate that the Raf-MEK-ERK signaling pathway plays a differential role during the time course of cardiac remodeling: increased phosphorylation and activation of ERK1/2 in early stage promotes cardiomyocyte hypertrophy and cardiac remodelling but in later stages decreased phosphorylation and downregulation of ERK1/2 increases myocyte apoptosis leading to LV dilatation and heart failure [14, 24–26]. Thus, systemic *RKIP*-deficiency ameliorates cardiac remodeling activating Raf-MEK-ERK signaling pathway during 5 weeks of pressure-overload.

TAC induced myocardial oxidative stress is a pivotal and well-characterized trigger of cardiac hypertrophy and fibrosis [18, 23, 45]. Nrf2 is the main transcriptional activator of antioxidant proteins and enzymes protecting against cardiac hypertrophy and fibrosis during pressure overload [7, 23, 45]. Immunostaining for 8-hydroxyguanosine revealed drastically increased oxidative stress both in pressure-overloaded cardiomyocytes and fibroblasts of WT N mice after TAC. Despite technical limitations of 8-hydroxyguanosine as oxidative damage marker [32] clinical studies demonstrate an increased level in patients with cardiovascular disease [8]. The finding of increased oxidative stress was confirmed by MDA concentration and decreased myocardial expressions of catalase and mitochondrial superoxide dismutase.

*RKIP*-knockout in N-mice markedly reduced oxidative stress and increased nuclear accumulation of Nrf2 [2]. This increase was accompanied by an enhanced myocardial protein expression of Keap1. Keap1 functions as substrate adaptor protein for Cullin 3(CUL3)/Ring box protein 1 (RBX1)-dependent E3 ubiquitin ligase complex which promotes rapid degradation of Nrf2 in the absence of oxidative stress [33]. Nrf2 regulates its protein level through an autoregulatory loop activating the expression of Keap1, CUL3 and RBX1 [33]. Keap1 decreases apoptosis and inflammation by reduction of NF-κB activation through Keap1E3 ligase-mediated degradation of IKKβ [6, 20]. The increased nuclear accumulation of Nrf2 is caused by down-regulated Jak2/Fyn myocardial signaling inhibiting export of Nrf2 from the nucleus (pathway depicted in the schematic (Fig. 8). *RKIP*^−*/*−^N mice demonstrate increased myocardial expression of GRK2 in pressure-overload and abrogated angiotensin II response in isolated adult cardiac fibroblasts [34]. We speculate that the ameliorative effect of Nrf2 in *RKIP*^−*/*−^ N TAC exceeds the deleterious one of GRK2 [5, 9, 39]. Moreover, ERK1 phosphorylates GRK2 reducing its pro-fibrotic activity [35]. Since RKIP is implicated in the regulation of different cell types we performed cell culture experiments to further confirm the influence of RKIP-knockout on the nuclear protein content of Nrf2 in adult cardiac fibroblasts. The fibroblasts from *RKIP*^−*/*−^ N mice demonstrate enhanced nuclear accumulation of Nrf2 after treatment with angiotensin II. Furthermore, small interfering RNA-mediated silencing of *RKIP* expression in adult cardiac fibroblasts of C57BL/6 N mice significantly reduced angiotensin II-induced expression of intracellular fibronectin and increased the nuclear accumulation of Nrf2. Our findings are in agreement with recent data from cell culture experiments demonstrating ameliorative effects of *RKIP* silencing: resistance to oxidative stress caused by increased nuclear accumulation of Nrf2 [2] and retardation of cellular senescence elicited by enhanced activity of ERK [22]. The retardation of aging can ameliorate cardiac fibrosis decreasing the number of cardiac myeloid and mesenchymal fibroblasts in age-dependent cardiac fibrosis [43]. These findings suggest that reduction of Ang II signaling leading to nuclear accumulation of Nrf2 represents an important mechanism of the observed ameliorative effects of systemic *RKIP*-deficiency in pressure-overloaded myocardium of C57BL/6 N mice.

### Effects of RKIP in C57BL/6-J mice

The *Nnt*-deficient C57BL/6 J strain is protected from myocardial oxidative stress in response to pressure overload [29]. Thus, we performed the proof-of concept experiments on *RKIP*^−*/*−^ J mice to elucidate the role of the myocardial redox status in the observed effects of *RKIP*-knockout. Overexpression *RKIP* is associated with increased cardiac contractility that is mediated by the β1-adrenoceptor and with anti-apoptotic and anti-fibrotic effects mediated by the β2-adrenoceptor (AR) [38]. The stimulation of β2-AR activates the cytoprotective antifibrotic and antiapoptotic phosphatidylinositol 3´kinase (PI3 K)-Akt signaling pathway [38, 40]. Systemic *RKIP*-deficiency in C57BL/6 J mice exaggerates pressure overload–induced cardiac failure [38]. Systemic *RKIP* deficiency has opposite effects on cardiac fibrosis in the pressure-overloaded myocardium of the two mouse strains: cardiac fibrosis and pro-fibrotic signaling were reduced in the *Nnt*-positive N and increased in the *Nnt*-negative J mice. Both basal and TAC-stimulated myocardial ROS production is markedly reduced in J mice. The expression of *PEBP*-*I*, the gene coding for RKIP, is higher in the N compared to the J mouse strain and TAC does not increase nuclear accumulation of Nrf2 in *RKIP*^−*/*−^ J mice. Thus, the fibrotic signaling of RKIP depends on the myocardial redox milieu. Under conditions of decreased myocardial ROS, detrimental effects of systemic *RKIP*-deficiency override its antioxidant protection.

To elucidate a potential clinical relevance of our findings, we evaluated the protein expression of RKIP, its phosphorylation level and nuclear accumulation of Nrf2 in the myocardial samples from non-failing and failing human left ventricles. The data suggest that RKIP protein expression correlates negatively with the nuclear protein content of Nrf2 in the human hearts. These findings are consistent with the concept that myocardial ROS production that is typical for maladaptive cardiac remodeling switches the effects of RKIP expression to pro-fibrotic signaling [4, 28]. Clearly, additional future studies are needed to confirm the role of RKIP during cardiac remodeling in humans.

Using immunohistochemistry to demonstrate the nuclear accumulation of Nrf2 has its limitations because of the potential non-specific cross-reactivity of some antibodies against Nrf2 [30, 31]. The association between the nuclear accumulation of Nrf2 and the reduced ROS production was reported previously, e.g. using Nrf2-LacZ mice [15] and GFP-Nrf2 fusion proteins [44]. Furthermore, antibody-based and antibody-independent detection of nuclear localization of Nrf2 revealed shuttling of Nrf2 between the nucleus and the cytoplasm [44].

In conclusion, the data show the important role of RKIP for the regulation of cardiac remodeling and fibrosis. Systemic RKIP deficiency ameliorates cardiac remodeling under conditions of increased myocardial production of reactive-oxygen species by activation of the Nrf2–Keap1 system. These findings may provide interesting perspectives to create novel strategies for the detection and prevention of myocardial fibrosis.

## Electronic supplementary material

Below is the link to the electronic supplementary material.

Supplementary material 1 (PDF 7755 kb)
