# Supplementary material for: Raf kinase inhibitor protein mediates myocardial fibrosis under conditions of enhanced myocardial oxidative stress
Source: Basic Res Cardiol. 2018 Sep 6;113(6):42. doi: 10.1007/s00395-018-0700-3 (PMC6133069; doi:10.1007/s00395-018-0700-3)
Supplement: Supplementary file 1 — Supplementary material 1 (PDF 7755 kb) [file 395_2018_700_MOESM1_ESM.pdf]

SUPPLEMENTAL MATERIAL ONLINE

Raf kinase inhibitor protein mediates myocardial fibrosis under conditions of enhanced myocardial oxidative stress

Basic Research in Cardiology

Andrey Kazakov[1] M.D., Rabea A. Hall[2], Christian Werner[1] M.D., Timo Meier[1] M.S., André Trouvain[1] M.S., Svetlana Rodionycheva[3] M.D., Frank Lammert[2] M.D., Christoph Maack[4] M.D., Michael Böhm[1] M.D., Ulrich Laufs[5] M.D.

[1]Klinik für Innere Medizin III, Kardiologie, Angiologie und Internistische Intensivmedizin, Universität/Universitätsklinikum des Saarlandes, Kirrberger Strasse 100, IMED, 66421 Homburg, Germany

[2]Klinik für Innere Medizin II, Gastroenterologie, Hepatologie, Endokrinologie, Diabetologie und Ernährungsmedizin, Universität/Universitätsklinikum des Saarlandes, Kirrberger Strasse, Gebäude 77, 66421 Homburg, Germany

[3]Klinik für Thorax- und Herz-Gefäßchirurgie, Universität/Universitätsklinikum des Saarlandes, Kirrberger Strasse 57, 66421 Homburg, Germany

[4]Deutsches Zentrum für Herzinsuffizienz, Universitätsklinikum Würzburg, am Schwarzenberg 15, A15, 97078 Würzburg, Germany

[5]Klinik und Poliklinik für Kardiologie, Universitätsklinikum Leipzig, Liebigstrasse 20, 04103 Leipzig, Germany

Running title: RKIP in myocardial oxidative stress and fibrosis

Correspondence to:
Dr. Andrey Kazakov
Klinik für Innere Medizin III, Kardiologie, Angiologie und Internistische Intensivmedizin
Universität/Universitätsklinikum des Saarlandes, 66424 Homburg/Saar, Germany
Tel.: +49-6841-16-15189
Fax: +49-6841-16-15175
E-Mail: Andrej.Kasakow@uks.eu
https://orcid.org/0000-0002-3031-0240

Supplemental Material and Methods

Animals and experimental design

Ten-week-old male C57BL/6N (N) albino wild-type (WT) mice and wild-type C57BL/6J (WT J) mice and *RKIP*$^{-/-}$ C57BL/6N albino mice (*RKIP*$^{-/-}$ N) mice and *RKIP*$^{-/-}$ C57BL/6J (*RKIP*$^{-/-}$ J) mice were housed under standard conditions. *RKIP*$^{-/-}$ N and *RKIP*$^{-/-}$J mice were backcrossed into the appropriate C57BL/6N or C57BL/6J background for more than 10 generations. The exons 7-11 of the gene encoding *nicotinamide nucleotide transhydrogenase (Nnt)* are missing in the C57BL/6J, but not C57BL/6N mice [1]. Due to a mutation of the *nicotinamide nucleotide transhydrogenase (Nnt)* gene the inbred mouse strain C57BL/6J is protected from oxidative stress in response to pressure overload [1]. Transverse aortic constriction (TAC) was performed to elicit replacement cardiac fibrosis. For surgery and left ventricle (LV)-pressure measurements animals were intraperitoneally anaesthetized with 100 mg/kg body weight ketaminehydrochloride (Ketanest®, Pfizer, Berlin, Germany) and 10 mg/kg body weight xylazinehydrochloride (Rompun® 2%, Bayer, Leverkusen, Germany). Anaesthetic monitoring was performed by testing of rear foot reflexes *before* and during procedures, observation of respiratory pattern, mucous membrane color, and responsiveness to manipulations throughout the procedures. After orotracheal intubation using a 20G catheter, the tube was connected to a volume cycled rodent ventilator (Harvard Apparatus, USA) on supplemental oxygen with a tidal volume of 0.2 mL and respiratory rate of 110 min^{-1}. The chest cavity was entered in the second intercostal space at the left upper sternal border through a small incision and aortic constriction was performed by tying a 7-0 nylon suture ligature against a 27G needle to yield a narrowing 360 µm in diameter and TAC of 65–70%. Sham-operated *RKIP*$^{-/-}$ N and WT N mice and unoperated *RKIP*$^{-/-}$ J and WT J were used as controls. After 5 weeks, LV-pressure measurements in *RKIP*$^{-/-}$ N and WT N sham- and TAC-mice were performed with 1.4 Fr pressure-transducing catheter (Mikro Tip Catheter, Millar instruments, USA). Experimental and control mice from our work group were sacrificed 24 hours, 1week and 5 weeks after surgery by i.p. injection of ketamine (1g/kg body weight) and xylazine (100 mg/kg) and hearts were rapidly excised. Hearts and other internal organs were partly snap-frozen in liquid nitrogen and stored at -80°C and partly embedded in paraffin after fixation in PBS-buffered formalin (4%). *RKIP*$^{-/-}$ J and WT J banded mice from the work group of the professor K. Lorenz were subjected to echocardiography 4 weeks after surgery [2]. Mice with an aortic pressure gradient higher than 60 mmHg and control unoperated mice were killed and their organ samples were collected as described above.

To induce interstitial cardiac fibrosis ten-week-old male and female WT N mice and *RKIP*$^{-/-}$ N mice were treated by intraperitoneal injections of carbon tetrachloride (Sigma-Aldrich, Germany) (0.7 mg CCl_4/kg body weight in mineral oil, final volume 50 µ l (Sigma-Aldrich, Germany) twice weekly for 6 weeks [3]. Forty-eight hours after the last injection the animals were anaesthetized and sacrificed as described above. Blood pressure was measured on the tail artery of mice 5 weeks after beginning of CCl_4 administration on 5 consecutive days using at least 20 repeating measurements per day (BP 2000 Series II Blood Pressure Analysis System, Visitech Systems, USA).

Flow cytometry for circulating fibroblasts (fibrocytes)

To obtain bone marrow cells mouse femur and tibia were flushed out several times with 5 ml of FACS-Buffer-BSA (containing 500ml of phosphate buffered saline [PBS], 25 ml fetal calf serum, 2.5 g bovine albumin, fraction V) using syringe with needle. The resulting cell suspension was filtered through a 70 µm cell strainer (BD Bioscience, Germany) in 15 ml conical tubes (PP-Test tubes; Greiner bio-one, Germany), and afterwards centrifuged at 1000 rpm, +4°C for 10 min with brakes switched off. Supernatant was aspirated with care because of the high vulnerability of bone marrow cells.

In FACS-tubes (BD Bioscience, Germany) with 100 µl of EDTA-blood or supernatant of bone marrow cells was added Lysing Buffer (BD Pharm Lyse, BD Bioscience, Germany) diluted 1:10 with distillated and deionized water to destroy erythrocytes. The tubes were vortexed and then left for 5 min on ice, followed by centrifugation at 1000 rpm for 10 min. Pellet was resuspended in 100 µl of FACS-

Buffer-BSA. Cells were permeabilized with Cytofix/Cytoperm (BD Bioscience, Germany) according to the manufacturer's instructions. To prevent non-specific binding of antibodies the pellet was resuspended in 300 µl of FACS-Buffer-BSA with 30 µl of anti CD16/32 antibody (BD Bioscience, Germany) and incubated in dark for 10 min at +4°C. After incubation 2 µl of the biotin-labeled antibody for Collagen I (Rockland, USA) were added to each tube. The tubes were vortexed and incubated in dark for 45 min at +4°C. To wash the cells 2 ml of FACS-Buffer-BSA were added to the suspension after which the tubes were centrifuged at 1750 rev./min, for 10 min. This procedure was repeated twice. After removal of the supernatant, the pellet was resuspended in 300 µl of FACS-Buffer-BSA with 10 µl of streptavidin-FITC (Vector Laboratories, USA) and incubated in dark for 30 min at +4°C. The wash step was repeated twice. The supernatant was resuspended in 300 µl of FACS-Buffer-BSA with 2 µl of the antibody for CD45 (BD Pharmingem APC-Cy7, Germany) and incubated in dark for 45 min at +4°C. The wash step was repeated twice. After removal of the supernatant, the pellet was resuspended in 0.2 ml of 1xPBS by vortexing. Isotype-identical antibodies served as controls (Becton Dickinson, Germany). The viable lymphocyte population was examined by flow cytometry (BD FACS CaliburTM instrument and BD CellQuest™ software) [4].

Isolation and cell culture of adult mouse cardiac fibroblasts

WT N and *RKIP*$^{-/-}$ N mice were anaesthetized with 5% Isofluran (Abbot, Germany). The removed heart was perfused in a retrograde perfusion system with a digestive solution containing Liberase TH® (Roche, Germany) for 6 min. The supernatant was centrifuged at 180g for 1 min. Collected pellet was transferred to culture dishes with Medium 199+GlutaMAX™ (Life Technologies, USA) supplemented with 10% fetal calf serum (PAA Laboratories, Austria) and HEPES (Life Technologies, USA) for 1h whereupon attached cells were supplemented with the fresh medium. Cells at 85% confluence in cell culture dishes were digested by trypsinization and replanted. Adult cardiac fibroblasts (ACF) from the second passage were used for all experiments. After 24 h in reduced serum medium, fibroblast cultures were treated with 1 µM angiotensin II (Sigma-Aldrich, Germany) for 5h. Cells were harvested for total protein isolation. To estimate extracellular matrix production western blot with an antibody for intracellular fibronectin (abcam, UK) was performed.

Migration assay

Adult cardiac fibroblasts were isolated and cultivated as described above. The second passage of ACF from WT and RKIP-/- C57BL/6N mice were treated with 1µM angiotensin II for 5 h or with 500 µM carbon tetrachloride dissolved in 0.1% dimethyl sulfoxide (DMSO) for 72 h then migration assay was performed as described. Untreated ACF or ACF treated with 0.1% DMSO were used as controls. To assess the migratory capacity, 500 fibroblasts from each culture dish were transferred to modified Boyden chambers (BD Bioscience, Germany) in plates filled with 750 µl medium containing 10 µL SDF-1 (R&D Systems, Germany) for 24 h whereupon were immunostained for intracellular fibronectin (abcam, UK). Boyden chamber filters were cut out, placed on slides and mounted with fluorescent mounting medium (Vectashield, Vector Laboratories, USA). The whole filter was analyzed using a Nikon E600 epifluorescence microscope (Nikon, Japan) with appropriate filters. Cells positive for fibronectin were counted.

Transfection experiments

Adult cardiac fibroblasts were isolated and cultivated as described above. Transfection of the second passage of ACF from WT C57BL/6N mice with siRNA *Pbp* (*RKIP*) 2 (SI01370194, Qiagen, USA) or with negative control (con) RNA (1022076, Qiagen, USA) using Lipofectamine RNAiMAX (13778-030, Invitrogen) and Opti-MEM reduced serum medium (31985-070, Life technologies, USA) was performed according to the manufacture instructions. After 24 hours of transfection ACF were treated with 1 µM angiotensin II (Sigma-Aldrich, Germany) for 5h. Untreated ACF were used as controls. Cells were harvested for preparation of nuclear and cytosolic protein fractures.

Immunofluorescence analysis

For pre-treatment slices with 3 µm thick deparaffinised sections were placed in Coplin jars with 0.05% citraconic anhydride solution, pH 7.4 for 1 hour at +98°C. Cultured cells were fixed for 20 min with 4% formaldehyde in 1xPBS. Tissue sections and fixed cells were incubated overnight at 4°C with the primary antibody, followed by the appropriate secondary antibody at 37°C for 1 hour. Immunofluorescence studies were performed by applying polyclonal antibodies against intracellular fibronectin (abcam, UK), 8-hydroxyguanosine (abcam, UK), CXCR4 (abcam, UK), PDGFRα (abcam, UK). Monoclonal antibody against α –sarcomeric actin (clone5c5, Sigma-Aldrich, Germany) was used to detect cardiomyocytes. Cycling cardiac cells were identified by immunostaining for proliferation marker Ki67 (abcam, UK). FITC-, TRITC–, and biotin-conjugated anti-mouse IgM, anti-mouse IgG, anti-rabbit IgG, and anti-goat IgG (all Dianova, Germany) were used as secondary antibodies and for amplification if necessary. After incubation with the biotinylated antibodies tissue sections were incubated with streptavidin-TRITC (Dianova, Germany) for 30 min at room temperature. To perform wash steps 4xSSC buffer was used. 4% bovine serum albumin in 4xSSC was used to prevent non-specific binding of antibodies. Sections were counterstained with DAPI (Calbiochem, Germany) and mounted with fluorescent mounting medium (Vectashield, Vector Laboratories, USA) for fluorescence microscopic analysis. All sections were blind and random evaluated using a Nikon E600 epifluorescence microscope (Nikon, Germany) with appropriate filters. In addition colocalization of stainings was controlled using a confocal microscopy unit (QLC100, VisiTech; United Kingdom on a Nikon E600 microscope).

Control of immunostainings

To exclude false positive and false negative results, all immunostainings were performed with appropriate positive and negative controls. As positive control for proliferation marker Ki-67 sarcoma sections were used. To control the specificity of the immunostaining for the intracellular fibronectin and PDGFRα immunostained sections were compared with the serial sections stained with picrosirius red. The purity of the fibroblast culture was confirmed by immunostainings which showed the presence of the fibroblast marker intracellular fibronectin (abcam, UK) and the absence of endothelial cell marker podocalyxin (R&D Systems, USA), inflammation cell marker CD45 (abcam, UK) and cardiomyocyte marker α-sarcomeric actin (Sigma-Aldrich, Germany).

Tissue morphometry

For morphometric analyses, LV tissue sections (3 µm) were blind and random examined. Cardiomyocyte cross sectional area was determined on photographs taken from hematoxylin and eosin (H&E) stained sections according to standard protocols at 400x magnification using a Nikon E600 microscope and the digital image software Lucia G (Nikon, Germany). The cardiomyocyte cross sectional area of at least 100 cardiomyocytes was measured in more than four different random areas of the section (1 section per heart).

The degree of cardiac fibrosis was determined by the use of picrosirius red staining. Formalin-fixed paraffin-embedded 3 µm thick heart sections were deparaffinised, immersed in picrosirius red solution for 1 hour at room temperature and washed in distilled water for 5 min. Thereafter the sections were dehydrated, cleared in xylene (Merck, Germany) and embedded in enthellan (Merck, Germany). The samples were examined and photographed at 100x magnification with a Nikon E600 microscope (Nikon, Germany). Ten fields from the subendocardial, the subepicardial and the middle part of the myocardium were evaluated. Analysis and quantification of total interstitial fibrillar collagen content was evaluated as fractional area of collagen content in % of myocardial tissue with the digital image software Lucia G (1 section per heart).

Numbers of fibroblasts, 8-hydroxyguanosine-positive cells and cardiomyocytes per mm^2 were determined by examining sections double stained for intracellular fibronectin, PDGFRα, 8-hydroxy-guanosine and α-sarcomeric actin respectively in 15 randomly chosen fields at 1000x magnification, (1 section per heart). Nuclei positive for intracellular fibronectin, 8-hydroxyguanosine and α-sarcomeric actin were counted. The cell density was calculated using the formula: cell density (mm^2)=cell number in 15 fields/((0.00153664)*15), where 0.00153664 is the area of the sampling grid at 1000x magnification, 15 is the number of counted fields .

Morphometric analysis for $Ki67^+$ and $CXCR4^+$ fibroblasts was performed on sections double-stained for these marker and intracellular fibronectin. The number of cycling cardiomyocytes was evaluated on sections double-stained for Ki67 and α-sarcomeric actin. In at least two sections from each mouse heart the total numbers of positive cells were evaluated by screening the total section area. In addition the size of the respective section area was determined by taking pictures at 20x magnification and pooling as well as evaluating them using Lucia G Software. From the total numbers of positive cells per area and from the numbers of fibroblasts and cardiomyocytes per area the percentages of Ki67 and CXCR4 expression in fibroblasts and expression of Ki67 in cardiomyocytes were calculated.

For the evaluation of the apoptotic indices, one section from each mouse heart was totally screened for apoptotic cardiomyocytes and non-cardiomyocytes. Apoptosis was detected using light field microscopy for the brown diaminobenzidine staining of the apoptosis kit; the co-immunostaining for the α-sarcomeric actin was evaluated by switching to the fluorescence unit of the same Nikon E600 microscope. After determining the total section area and the numbers of the specific cells as described above, we were able to calculate apoptotic indices as percentage of apoptotic cells in the respective evaluated group.

Gene expression analysis

Gene expression was measured by the semi-quantitative reverse transcriptase-polymerase chain reaction (RT-PCR) and the real-time quantitative RT-PCR using the TaqMan system (AB Step One Plus, Applied Biosystems, Germany). Briefly, RNA from the LV myocardium was extracted using the peqGOLD RNAPure (Peqlab Biotechnologie, Germany). Oligo (dT) primed cDNA synthesis was performed with High capacity cDNA reverse transcription kit (Thermo Fisher Scientific, Germany). Homozygous deletion and expression of *PEBP-1* were elicited using the following primers: forward primer 5′ GCTTTCCAGGCCTCTCAGTGTTCATCAG 3′; reverse primer: 5′ CCCGCCCATCCTGCCCATAGG 3′.[2] *GAPDH* was amplified as the external standard. Each PCR cycle consisted of denaturing at 95°C for 45 s, annealing at 62°C, 35 cycles for *Pebp1* and at 55°C 30 cycles for *GAPDH*. Equal amounts of *Pebp1* and *GAPDH* RT-PCR products were loaded on 1.5% agarose gels, and optical densities of ethidium bromide-stained DNA bands were quantified. Expression of *collagen Iα2* (Mm01165187_m1), *CTGF*, (Mm01192931_g1) and *hypoxanthine-guanine phosphoribosyltransferase (HGPRT)* (Mm 01545399_m1) was analyzed with TaqMan gene expression assays-on-demand purchased from Thermo Fisher Scientific, Germany. For quantification, mRNA amount of the respective gene was normalized to the amount of *HGPRT* using the $2^{-\Delta\Delta CT}$ method.

Western blot analysis

For preparation of whole protein extract, heart tissue and cultured cells were homogenized, dissolved in lysis buffer (containing 20mM 4-(2-hydroxyethyl)-1-piperazineethanesulfonic acid [HEPES] pH7.4, 2mM ethylene glycol tetraacetic acid [EGTA], 1mM dithiothreitol [DTT], 1mM sodium orthovanadate [Na_3VO_4], 1% triton X-100, 10% glycerol, 2 µM leupeptin, 0.4 mM phenylmethanesulphonylfluoride [PMSF] and 10 µg/ml aprotinin, 2 µM microcystin, 50 µM ß-glycerolphosphate), centrifuged for 3 min, 10000 rev./min, +4 °C and stored at –80 °C.

For preparation of nuclear protein fraction, heart tissue and cultured cells were homogenized in puffer A (containing 10mM HEPES pH7.9, 10mM potassium chloride [KCl], 0.1mM ethylene diamine

tetraacetic acid [EDTA], 0.1mM EGTA, 1mM DTT, 0.5mM PMSF), incubated for 30 min on ice, mixed with 10% IGEPAL (Sigma-Aldrich, Germany), centrifuged for 1 min, 13000 rev./min. Pellet was dissolved in puffer C (containing 20mM HEPES pH7.9, 0.4M sodium chloride [NaCl], 1mM EDTA, 1mM EGTA, 1 mM DTT, 1mM PMSF), incubated on ice for 15 min, mixed and centrifuged for 1 min, 13000 rev./min. Supernatant with nuclear protein fracture was stored at –80 °C.

Heart tissue lysate was immunoprecipitated using anti-Jak2 antibodies (Cell Signaling Technology, USA) plus protein A sepharose CL-4B (GE Healthcare, Germany). Isolated proteins were separated by 12% SDS-PAGE and transferred to nitrocellulose membranes (Bio-Rad Laboratories, Germany). Non-specific protein was blocked with 5% dried milk for 1 h and the membranes were incubated overnight at 4°C with primary antibody against RKIP (abcam, UK), Ser153 phospho-RKIP (Santa-Cruz Biotechnology, USA), intracellular fibronectin (abcam, UK), nuclear factor erythroid 2-related factor 2 (Nrf2) (abcam, UK), Kelch-like ECH-associated protein 1 (Keap1) (abcam, UK), Fyn (Cell Signaling Technology, USA), Thr12 phospho-Fyn (Santa-Cruz Biotechnology, USA), Jak2 (Cell Signaling Technology, USA), Tyr 1007/1008 phospho-Jak2 (Cell Signaling Technology, USA), mRNA-polymerase II (Merck Millipore, Germany), extracellular signal-regulated kinases (ERK) and phospho-ERK (Cell Signaling Technology, USA), catalase (Cell Signaling Technology, USA), mitochondrial superoxide dismutase/superoxide dismutase 2 (Santa-Cruz Biotechnology, USA), and GAPDH (Santa-Cruz Biotechnology, USA) and at the next day with appropriate horseradish peroxidase-conjugated secondary antibodies for 20 min at room temperature in phosphate-buffered saline. Visualization was performed with an enhanced chemiluminescence kit (GE Healthcare, UK). Autoradiography was carried out at 23 °C, and the appropriate exposures were quantitated by densitometry.

Malondialdehyde concentrations

Lipid peroxidation was performed using the ALDetect Lipid Peroxidation Assay Kit (Enzo Life Science, Germany) to detect the concentrations of malondialdehyde (MDA) according to the manufacturer´s instructions [1].

Supplemental Figure Legends

Suppl. Fig. 1 Systemic *RKIP*-deficiency reduces mRNA-expression of *collagen Iα2* and *connective tissue growth factor (CTGF)* in CCl_4-treated and pressure-overloaded LV myocardium of *RKIP-/-* N mice

Collagen Iα2- mRNA (a) and *CTGF*-mRNA expression (b) in experimental and control groups. One-way ANOVA with Fisher LSD post hoc test.

Suppl. Fig. 2 Systemic *RKIP*-deficiency reduces cardiomyocyte apoptosis in CCl_4-treated and *RKIP-/-* TAC N

RKIP-knockout diminished the percentage of cardiomyocyte apoptosis in N CCl_4-treated and N TAC mice (a) and reduced the percentage of apoptotic non-cardiomyocytes in N TAC mice (b) (n=10 per group). Representative sections of the LV myocardium show light microscopic staining for apoptosis (brown) and fluorescence microscopy for the myocyte marker α–sarcomeric actin (green) (c). Apoptotic nuclei are marked by arrowheads. Nuclei are stained blue by DAPI. Bars = 30 µm. One-way ANOVA with Fisher LSD post hoc test.

Suppl. Fig. 3 Systemic *RKIP*-deficiency reduces fibroblast apoptosis in CCl_4-treated and *RKIP-/-* TAC N

RKIP-knockout diminished the percentage of fibroblast apoptosis in N CCl_4-treated and N TAC mice (n=3 per group) (a). Representative sections of the LV myocardium show light microscopic staining for apoptosis (brown) and fluorescence microscopy for the myocyte marker α–sarcomeric actin (red) and the fibroblast marker intracellular fibronectin (green) (b). Apoptotic nuclei of fibroblasts are marked by arrowheads. Nuclei are stained blue by DAPI. Bars = 30 µm. One-way ANOVA with Fisher LSD post hoc test.

Suppl. Fig. 4 *RKIP*-knockout reduces the percentage of cycling $Ki67^+$cardiac fibroblasts and $Ki67^+$cardiomyocytes in both models of cardiac fibrosis

RKIP-knockout reduced the percentage of cycling $Ki67^+$ fibroblasts (a) and $Ki67^+$cardiomyocytes (b) in CCl_4-treated and *RKIP-/-* N TAC mice (n=9-10 per group). (c) $Ki67^+$ cardiomyocytes (marked by arrowheads) and $Ki67^+$non-cardiomyocyte (marked by arrow) in a LV section from a WT N TAC mouse: α–sarcomeric actin (green), Ki67 (red), nuclei stained blue by DAPI and the overlay of the three stainings. Bars = 10 µm. One-way ANOVA with Fisher LSD post hoc test.

Suppl. Fig. 5 Different expression of the active phosphorylated form of ERK 1/2 in the time course of pressure overload in WT N and *RKIP-/-* N mice

Both *RKIP*-knockout and 1 day-TAC (n=3-6 per group) (a), increased the phosphorylation of Thr202/Tyr204-ERK1/2 which remained unchanged during the first week after surgery (n=4 per group) (b) but was significantly reduced in WT N TAC 5 weeks later (n=3-4 per group) (c). One-way ANOVA with Fisher LSD post hoc test.

Suppl. Fig. 6 Systemic *RKIP*-deficiency leads to increased nuclear localization of Nrf2 in pressure overloaded cardiomyocytes and fibroblasts of $RKIP^{-/-}$ N mice

Pressure-overloaded $RKIP^{-/-}$ N mice demonstrated increased nuclear localization of Nrf2 in cardiomyocytes and cardiac fibroblasts. (a) Representative images of cardiomyocytes in LV sections: myocytic α-sarcomeric actin (green), Nrf2 (red), nuclei stained blue by DAPI, the overlay of the three stainings and image of cardiomyocyte nuclei marked by arrowheads. (b) Representative images of

fibrotic area in LV sections: intracellular fibronectin (green), Nrf2 (red), nuclei stained blue by DAPI, the overlay of the three stainings and image of fibroblast nuclei marked by arrowheads. Bars = 10 µm.

Supplemental Table 1 Antibodies

Antibody	Company	Catalog #	Application	Working Dilution
Anti-CD16/CD32 Mouse BD Fc Block^{TM}	BD Bioscience	553142	Flow Cytometry	1:11
Anti-Collagen I-Biotin-conjugated	Rockland	600-406-103	Flow Cytometry	1:165
Anti-Mouse CD45-APC-Cy7	BD Bioscience	557659	Flow Cytometry	1:151
Biotin-SP-conjugated ChromPure Rabbit-IgG whole molecule Isotype control	Dianova	011-060-003	Flow Cytometry	1:165
APC Rat IgG2b k Isotype Control	BD Bioscience	556924	Flow Cytometry	1:151
Rabbit anti intracellular fibronectin	abcam	ab23750	Immunofluorescence<br>Western Blot	1:300<br>1:5000
Mouse anti intracellular fibronectin	abcam	ab6328	Immunofluorescence	1:50
PDGFRalpha	abcam	ab51875	Immunofluorescence	1:30
Rabbit anti ki67	abcam	ab15580	Immunofluorescence	1:200
Goat anti 8-hydroxy-guanosin	abcam	ab10802	Immunofluorescence	1:300
Goat anti CXCR4	abcam	ab1670	Immunofluorescence	1:50
Mouse anti α-sarcomeric actin	Sigma-Aldrich	A2172	Immunofluorescence	1:100
Rabbit anti CD45	abcam	ab10558	Immunofluorescence	1:100
Goat anti podocalyxin	RD Systems	AF1556	Immunofluorescence	1:30
Anti rabbit IgG biotin	Dianova	711-065-152	Immunofluorescence	1:30
Anti goat IgG biotin	Dianova	705-065-147	Immunofluorescence	1:30
Anti rabbit IgG FITC	Dianova	711-095-152	Immunofluorescence	1:50
Anti mouse IgG FITC	Dianova	715-095-150	Immunofluorescence	1:50
Anti rabbit IgG TRITC	Dianova	711-025-152	Immunofluorescence	1:50

Anti mouse IgM FITC	Dianova	115-097-020	Immunofluorescence	1:50
Anti rat IgG FITC	Dianova	112-095-167	Immunofluorescence	1:30
Rabbit anti phospho- RKIP (Ser 153)	Santa Cruz Biotechnology	sc-32623	Western Blot	1:250
Rabbit anti RKIP	abcam	ab76582	Western Blot	1:1000
Rabbit anti Nrf2	abcam	ab31163	Western Blot, Immunofluorescence	1:500 1:30
Rabbit anti Keap1	abcam	ab66620	Western Blot	1:500
Mouse anti GRK2	Santa Cruz Biotechnology	sc-13143	Western Blot	1:250
Mouse anti RNA Polymerase II	Millipore	05-623	Western Blot	1:4000
Mouse anti GAPDH	Millipore	MAB 374	Western Blot	1:10 000
Rabbit anti phospho-p44/42 MAPK (ERK1/2) (Thr202/Tyr204)	Cell Signaling Technology	9101	Western Blot	1:1000
Rabbit anti p44/42 MAPK (Erk1/2)	Cell Signaling Technology	9102	Western Blot	1:1000
Mouse anti phospho-Fyn (Thr12)	Santa Cruz Biotechnology	sc-377555	Western Blot	1:250
Rabbit anti Fyn	Cell Signaling Technology	4023	Western Blot	1:1000
Rabbit anti phospho-Jak2 (Tyrb1007/1008)	Cell Signaling Technology	3776	Western Blot	1:1000
Rabbit anti Jak2	Cell Signaling Technology	3230	Western Blot	1:1000
Catalase	Cell Signaling Technology	14097	Western Blot	1:1000
Mitochondrial superoxide dismutase /superoxide dismutase 2	Santa Cruz Biotechnology	sc-30080	Western Blot	1:1000
Anti rabbit IgG Peroxidase	Bio-Rad Laboratories	172-1019	Western Blot	1:5000
Anti mouse IgG Peroxidase	Bio-Rad Laboratories	170-6516	Western Blot	1:5000

References

1. Nickel AG, von Hardenberg A, Hohl M, Löffler JR, Kohlhaas M, Becker J, Reil JC, Kazakov A, Bonnekoh J, Stadelmaier M, Puhl SL, Wagner M, Bogeski I, Cortassa S, Kappl R, Pasieka B, Lafontaine M, Lancaster CRD, Blacker TS, Hall AR, Duchen MR, Kästner L, Lipp P, Zeller T, Müller C, Knopp A, Laufs U, Böhm M, Hoth M, Maack C (2015) Reversal of mitochondrial transhydrogenase causes oxidative stress in heart failure. Cell Metab 22:472-484. doi: 10.1016/j.cmet.2015.07.008
2. Schmid E, Neef S, Berlin C, Tomasovic A, Kahlert K, Nordbeck P, Deiss K, Denzinger S, Herrmann S, Wettwer E, Weidendorfer M, Becker D, Schäfer F, Wagner N, Ergün S, Schmitt JP, Katus HA, Weidemann F, Ravens U, Maack C, Hein L, Ertl G, Müller OJ, Maier LS, Lohse MJ, Lorenz K (2015) Cardiac RKIP induces a beneficial β-adrenoceptor-dependent positive inotropy. Nat Med 21:1298-1306. doi: 10.1038/nm.3972
3. Hall RA, Liebe R, Hochrath K, Kazakov A, Alberts R, Laufs U, Böhm M, Fischer HP, Williams RW, Schughart K, Weber SN, Lammert F (2014) Systems genetics of liver fibrosis: identification of fibrogenic and expression quantitative trait loci in the BXD murine reference population. PLoS One 9:e89279. doi: 10.1371/journal.pone.0089279
4. Kazakov A, Hall R, Jagoda P, Bachelier K, Müller-Best P, Semenov A, Lammert F, Böhm M, Laufs U (2013) Inhibition of endothelial nitric oxide synthase induces and enhances myocardial fibrosis. Cardiovasc Res100:211-221. doi: 10.1093/cvr/cvt181

Suppl. Fig. 1

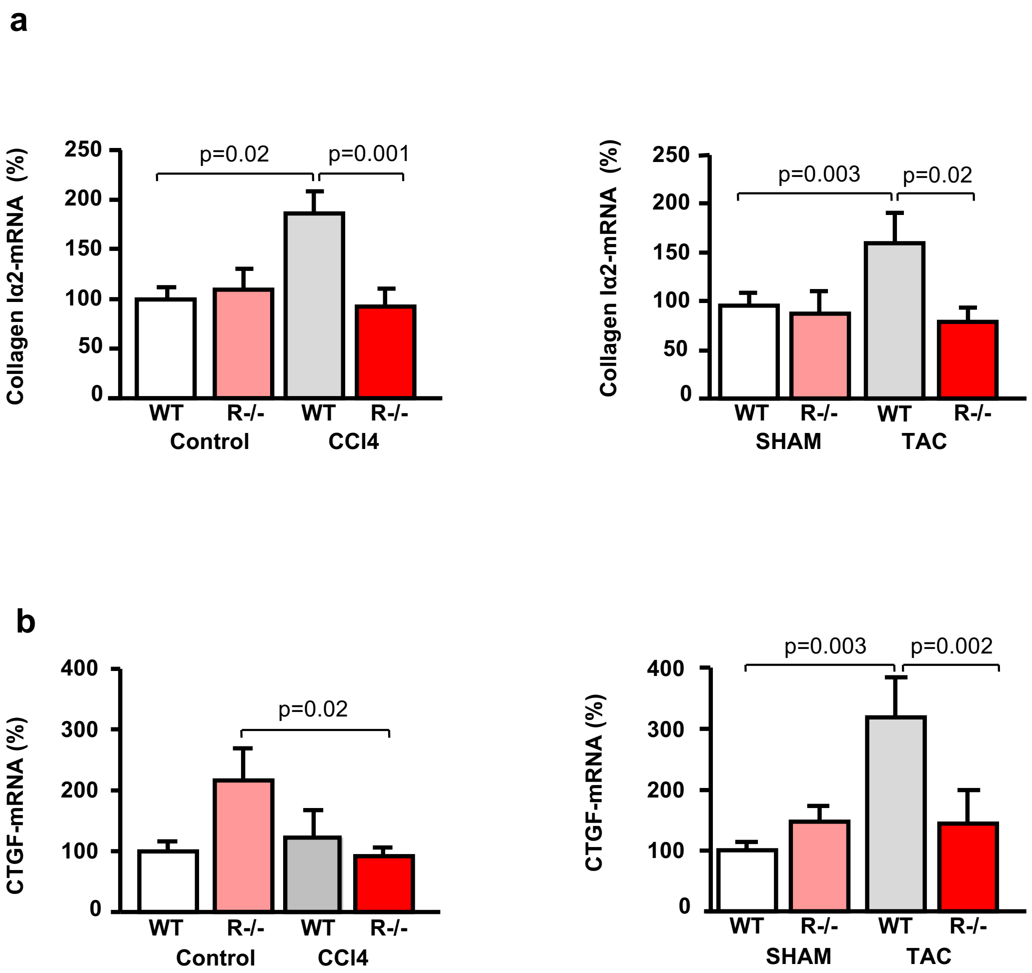
a
Collagen Iα2-mRNA (%)
250
200
150
100
50
0
p=0.02
p=0.001
WT
R-/-
WT
R-/-
Control
CCl4
p=0.003
p=0.02
SHAM
TAC
b
CTGF-mRNA (%)
400
300
200
100
0
p=0.02
p=0.003
p=0.002

Suppl. Fig. 2

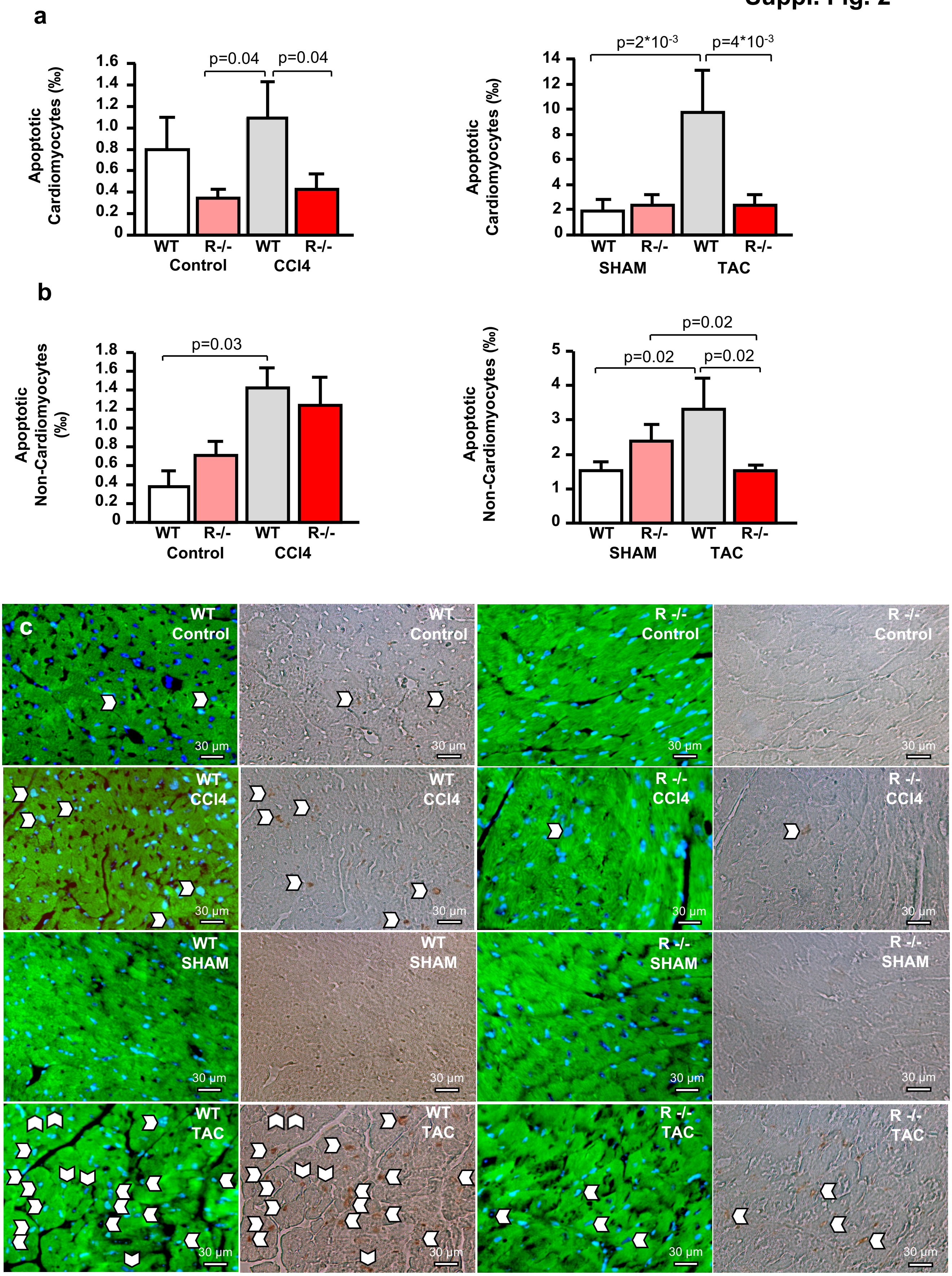
a
Apoptotic Cardiomyocytes (‰)
p=0.04
p=0.04
WT
R-/-
Control
CCl4
p=2*10-3
p=4*10-3
SHAM
TAC
b
Apoptotic Non-Cardiomyocytes (‰)
p=0.03
p=0.02
p=0.02
p=0.02
c
WT Control
R -/- Control
WT CCl4
R -/- CCl4
WT SHAM
R -/- SHAM
WT TAC
R -/- TAC
30 µm

Suppl. Fig. 3

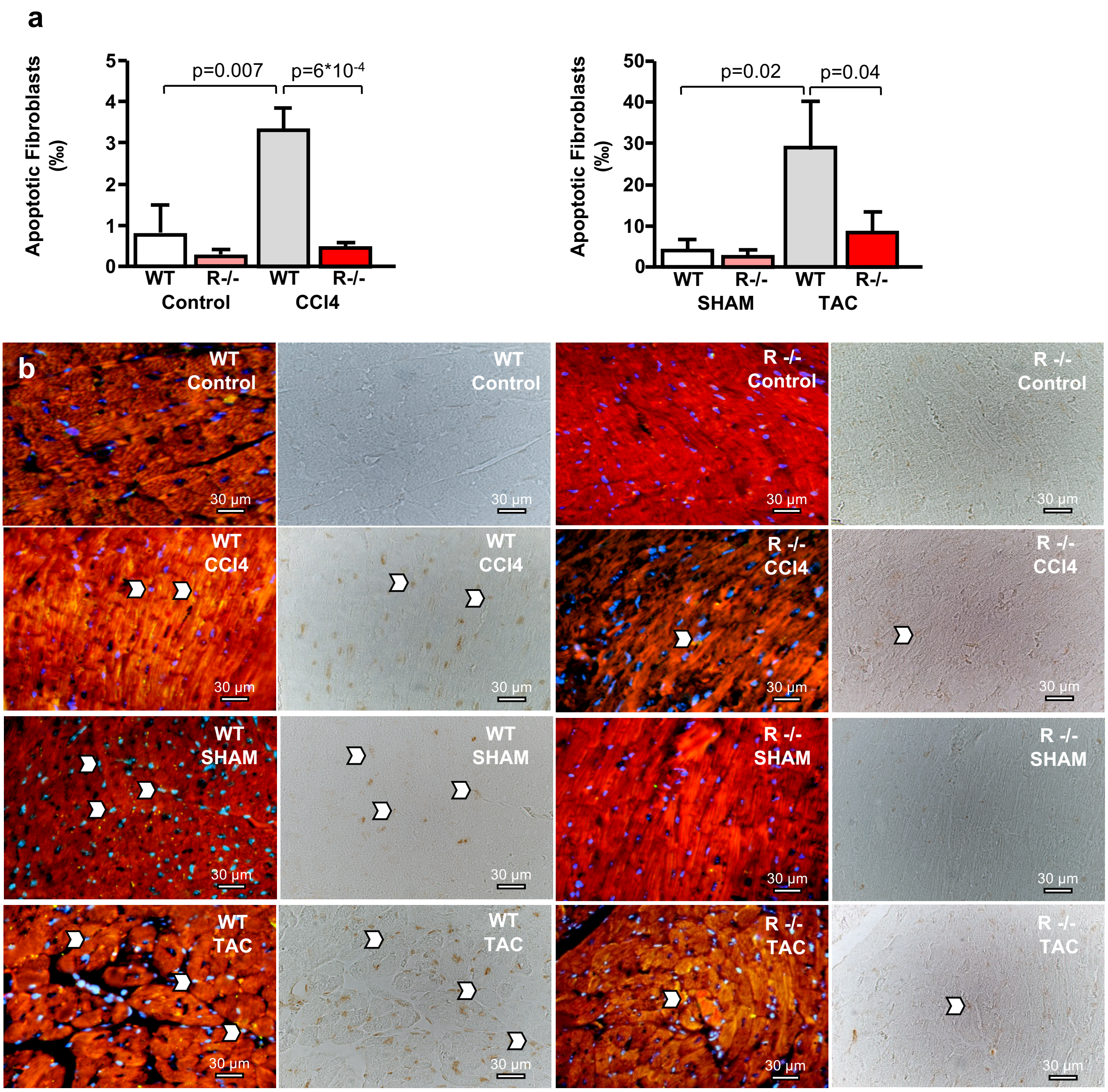
a
Apoptotic Fibroblasts (‰)
p=0.007
p=6*10-4
WT
R-/-
WT
R-/-
Control
CCl4
p=0.02
p=0.04
SHAM
TAC
b
WT Control
R -/- Control
WT CCl4
R -/- CCl4
WT SHAM
R -/- SHAM
WT TAC
R -/- TAC
30 µm

Suppl. Fig. 4

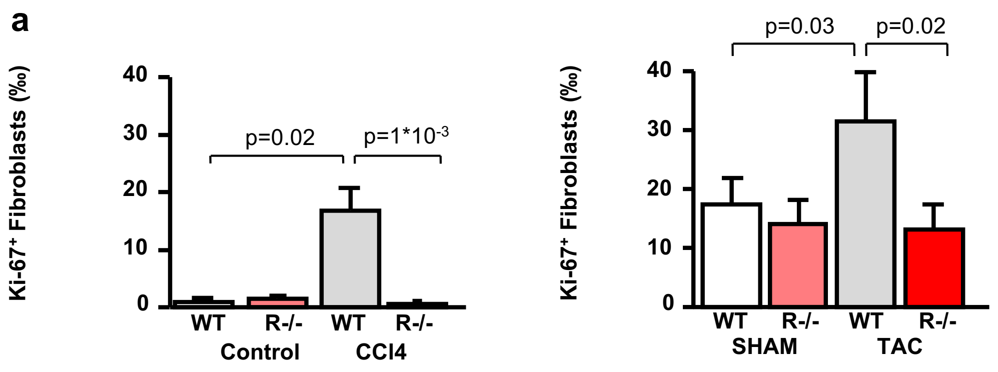

a
Ki-67+ Fibroblasts (‰)
40
30
20
10
0
p=0.02
p=1*10-3
WT
R-/-
WT
R-/-
Control
CCl4
Ki-67+ Fibroblasts (‰)
p=0.03
p=0.02
WT
R-/-
WT
R-/-
SHAM
TAC

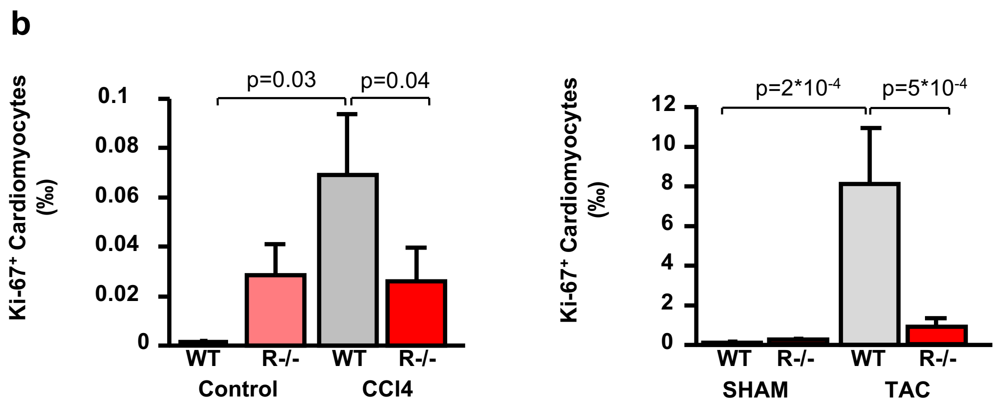

b
Ki-67+ Cardiomyocytes (‰)
0.1
0.08
0.06
0.04
0.02
0
p=0.03
p=0.04
WT
R-/-
WT
R-/-
Control
CCl4
12
10
8
6
4
2
0
p=2*10-4
p=5*10-4
SHAM
TAC

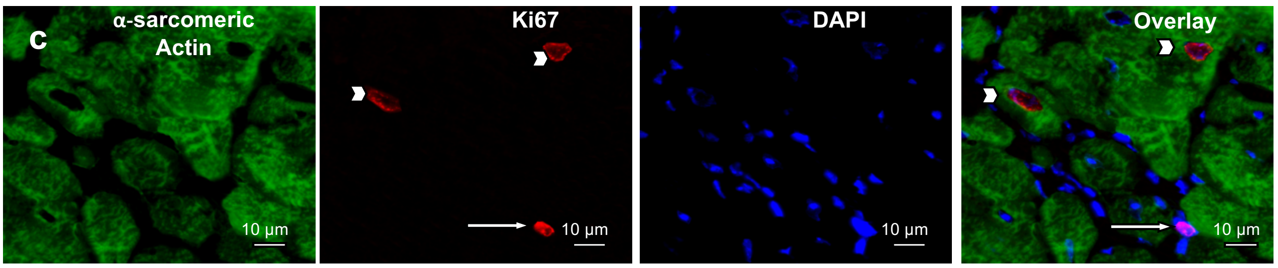

c
α-sarcomeric Actin
Ki67
DAPI
Overlay
10 µm

Suppl. Fig. 5

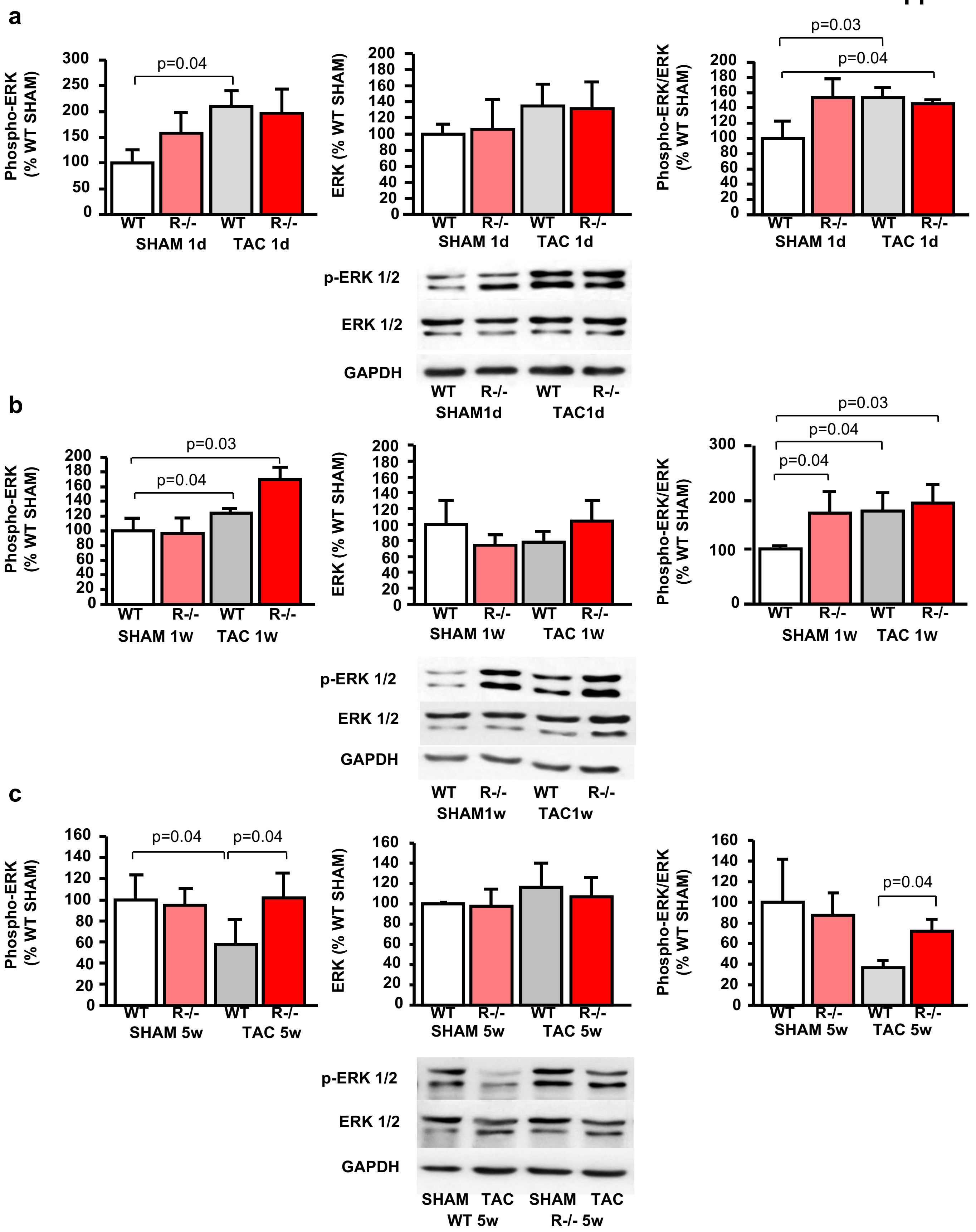

a
Phospho-ERK (% WT SHAM)
p=0.04
WT R-/- WT R-/-
SHAM 1d TAC 1d
ERK (% WT SHAM)
Phospho-ERK/ERK (% WT SHAM)
p=0.03
p=0.04
p-ERK 1/2
ERK 1/2
GAPDH
SHAM1d TAC1d
b
p=0.03
p=0.04
SHAM 1w TAC 1w
SHAM1w TAC1w
c
p=0.04
SHAM 5w TAC 5w
SHAM TAC SHAM TAC
WT 5w R-/- 5w

Suppl. Fig. 6

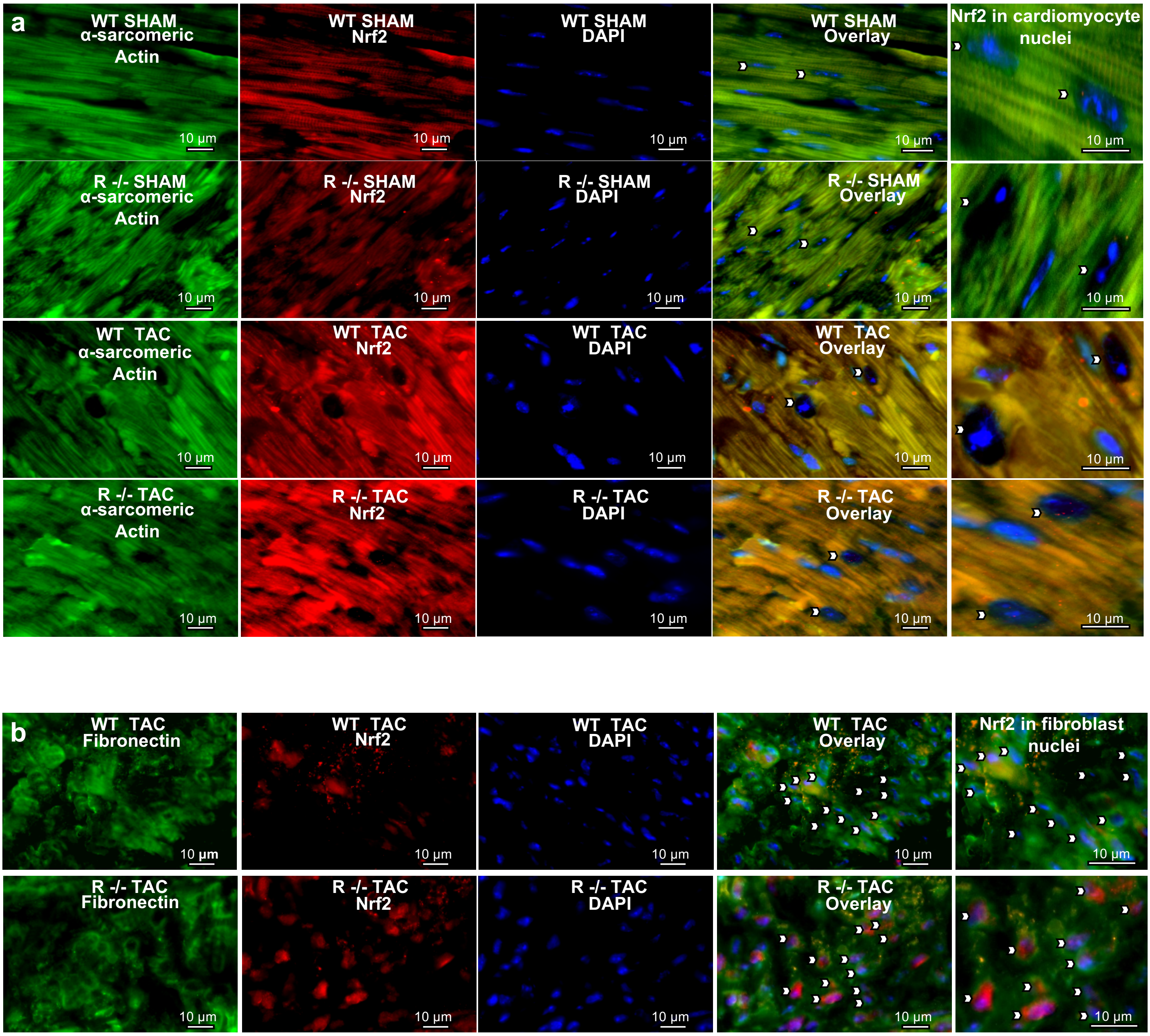

a
WT SHAM α-sarcomeric Actin
WT SHAM Nrf2
WT SHAM DAPI
WT SHAM Overlay
Nrf2 in cardiomyocyte nuclei
R -/- SHAM α-sarcomeric Actin
R -/- SHAM Nrf2
R -/- SHAM DAPI
R -/- SHAM Overlay
WT TAC α-sarcomeric Actin
WT TAC Nrf2
WT TAC DAPI
WT TAC Overlay
R -/- TAC α-sarcomeric Actin
R -/- TAC Nrf2
R -/- TAC DAPI
R -/- TAC Overlay
10 µm
b
WT TAC Fibronectin
WT TAC Nrf2
WT TAC DAPI
WT TAC Overlay
Nrf2 in fibroblast nuclei
R -/- TAC Fibronectin
R -/- TAC Nrf2
R -/- TAC DAPI
R -/- TAC Overlay
10 µm
